# Genetic Determinants of Circulating Estrogen Levels and Evidence of a Causal Effect of Estradiol on Bone Density in Men

**DOI:** 10.1210/jc.2017-02060

**Published:** 2018-01-09

**Authors:** Anna L Eriksson, John R B Perry, Andrea D Coviello, Graciela E Delgado, Luigi Ferrucci, Andrew R Hoffman, Ilpo T Huhtaniemi, M Arfan Ikram, Magnus K Karlsson, Marcus E Kleber, Gail A Laughlin, Yongmei Liu, Mattias Lorentzon, Kathryn L Lunetta, Dan Mellström, Joanne M Murabito, Anna Murray, Maria Nethander, Carrie M Nielson, Inga Prokopenko, Stephen R Pye, Leslie J Raffel, Fernando Rivadeneira, Priya Srikanth, Lisette Stolk, Alexander Teumer, Thomas G Travison, André G Uitterlinden, Dhananjay Vaidya, Dirk Vanderschueren, Joseph M Zmuda, Winfried März, Eric S Orwoll, Pamela Ouyang, Liesbeth Vandenput, Frederick C W Wu, Frank H de Jong, Shalender Bhasin, Douglas P Kiel, Claes Ohlsson

**Affiliations:** 1 Centre for Bone and Arthritis Research, Institute of Medicine, Sahlgrenska University Hospital, Gothenburg, Sweden; 2 Medical Research Council Epidemiology Unit, University of Cambridge School of Clinical Medicine, Institute of Metabolic Science, Cambridge Biomedical Campus, Cambridge, United Kingdom; 3 University of Exeter Medical School, University of Exeter, Exeter, United Kingdom; 4 Duke University School of Medicine, Durham, North Carolina; 5 Vth Department of Medicine, Medical Faculty Mannheim, Heidelberg University, Mannheim, Germany; 6 Longitudinal Studies Section, Clinical Research Branch, Gerontology Research Center, National Institute on Aging, Baltimore, Maryland; 7 Division of Endocrinology, Stanford University School of Medicine, Stanford, California; 8 Department of Surgery and Cancer, Imperial College London, Hammersmith Campus, London, United Kingdom; 9 Department of Physiology, Institute of Biomedicine, University of Turku, Turku, Finland; 10 Department of Epidemiology, Erasmus MC, Rotterdam, The Netherlands; 11 Department of Orthopaedics and Clinical Sciences, Skåne University Hospital, Lund University, Malmö, Sweden; 12 Family Medicine and Public Health, University of California-San Diego, San Diego, California; 13 Department of Epidemiology and Prevention, Division of Public Health Sciences, Wake Forest School of Medicine, Winston-Salem, North Carolina; 14 Geriatric Medicine, Department of Internal Medicine and Clinical Nutrition, Institute of Medicine, University of Gothenburg and Geriatric Medicine, Sahlgrenska University Hospital, Mölndal, Sweden; 15 Boston University School of Public Health, Boston, Massachusetts; 16 Framingham Heart Study, Framingham, Massachusetts; 17 Department of Medicine, Section of General Internal Medicine, Boston University School of Medicine, Boston, Massachusetts; 18 School of Public Health, Oregon Health & Science University, Portland, Oregon; 19 Department of Genomics of Common Disease, School of Public Health, Imperial College London, London, United Kingdom; 20 Hammersmith Hospital, London, United Kingdom; 21 Arthritis Research UK Centre for Epidemiology, Centre for Musculoskeletal Research, The University of Manchester, Manchester Academic Health Science Centre, Manchester, United Kingdom; 22 Division of Genetic and Genomic Medicine, Department of Pediatrics, University of California, Irvine, California; 23 Department of Internal Medicine, Erasmus MC, Rotterdam, The Netherlands; 24 Institute for Community Medicine, University Medicine Greifswald, Greifswald, Germany; 25 Interfaculty Institute for Genetics and Functional Genomics, University Medicine Greifswald, Greifswald, Germany; 26 Institute for Aging Research, Hebrew Senior Life and Department of Medicine, Beth Israel Deaconess Medical Center and Harvard Medical School, Boston, Massachusetts; 27 Department of Medicine, Johns Hopkins University School of Medicine, Baltimore, Maryland; 28 Department of Clinical and Experimental Medicine, Katholieke Universiteit Leuven, Laboratory of Clinical and Experimental Endocrinology, Leuven, Belgium; 29 Department of Epidemiology, University of Pittsburgh, Pittsburgh, Pennsylvania; 30 Synlab Academy, Synlab Holding Deutschland GmbH, Mannheim, Germany; 31 Clinical Institute of Medical and Chemical Laboratory Diagnostics, Medical University of Graz, Graz, Austria; 32 Bone & Mineral Unit, Oregon Health & Science University, Portland, Oregon; 33 Andrology Research Unit, Centre for Endocrinology and Diabetes, Institute of Human Development, Faculty of Medical and Human Sciences, The University of Manchester, Central Manchester University Hospitals National Health Service Foundation Trust, Manchester, United Kingdom; 34 Research Program in Men's Health: Aging and Metabolism, Brigham and Women's Hospital, Harvard Medical School, Boston, Massachusetts

## Abstract

**Context:**

Serum estradiol (E2) and estrone (E1) levels exhibit substantial heritability.

**Objective:**

To investigate the genetic regulation of serum E2 and E1 in men.

**Design, Setting, and Participants:**

Genome-wide association study in 11,097 men of European origin from nine epidemiological cohorts.

**Main Outcome Measures:**

Genetic determinants of serum E2 and E1 levels.

**Results:**

Variants in/near *CYP19A1* demonstrated the strongest evidence for association with E2, resolving to three independent signals. Two additional independent signals were found on the X chromosome; *FAMily with sequence similarity 9, member B (FAM9B)*, rs5934505 (*P* = 3.4 × 10^−8^) and *Xq27.3*, rs5951794 (*P* = 3.1 × 10^−10^). E1 signals were found in *CYP19A1* (rs2899472, *P* = 5.5 × 10^−23^), in *Tripartite motif containing 4 (TRIM4*; rs17277546, *P* = 5.8 × 10^−14^), and *CYP11B1/B2* (rs10093796, *P* = 1.2 × 10^−8^). E2 signals in *CYP19A1* and *FAM9B* were associated with bone mineral density (BMD). Mendelian randomization analysis suggested a causal effect of serum E2 on BMD in men. A 1 pg/mL genetically increased E2 was associated with a 0.048 standard deviation increase in lumbar spine BMD (*P* = 2.8 × 10^−12^). In men and women combined, *CYP19A1* alleles associated with higher E2 levels were associated with lower degrees of insulin resistance.

**Conclusions:**

Our findings confirm that *CYP19A1* is an important genetic regulator of E2 and E1 levels and strengthen the causal importance of E2 for bone health in men. We also report two independent loci on the X-chromosome for E2, and one locus each in *TRIM4* and *CYP11B1/B2*, for E1.

## Teaser

CYP19A1 was the main regulator of estrogen levels in this GWAS, with additional loci on chromosome X and in TRIM4 and CYP11B1/B2. Findings in the study strengthen the importance of E2 for bone health.

Estrogens 17 *β*-estradiol (E2) and estrone (E1) are the major biologically active estrogens in men. E2 is more potent than E1. Aromatase, encoded by the *CYP19A1* gene, is the key enzyme responsible for the final step in the synthesis of both E2 and E1. E2 is formed from aromatization of testosterone, and E1 is formed from aromatization of androstenedione. E2 can also be formed from conversion of E1 by 17*β*-hydroxysteroid dehydrogenase (1).

In men, the circulating levels of E2 and E1 are determined by both genetic and environmental factors. The heritability for E2 in men has been estimated to be ∼30% to 45% and for E1 ∼40% (2, 3). Early studies of the genetic regulation of circulating E2 and E1 levels were hampered by their small size and the use of immunoassays with poor specificity, precision, and accuracy at lower concentrations. However, in 2010, Orwoll and colleagues performed a large study of 5000 elderly men of European, Asian, and African origin in Sweden, the United States, Hong Kong, and Tobago (4). Serum sex steroid levels were measured using gas chromatography-mass spectrometry (GC-MS), thereby avoiding the previously mentioned problems with immunoassays. In addition to geographical differences in E2 and E1 levels, suggestive of environmental influences, they also found racial differences. Both E2 and E1 levels, as well as the E2 to testosterone and E1 to androstenedione ratios, were higher in black than in Asian and Caucasian men (4). These data suggested that genetically determined differences in aromatase activity among black, Asian, and Caucasian men might be responsible for the observed racial differences in E2 and E1 levels.

We made a first attempt to find genetic loci involved in the determination of estrogen levels in men by analyzing 604 single nucleotide polymorphisms (SNPs) in 50 candidate sex steroid-related genes (5). In a screening cohort, the *CYP19A1* SNP rs2470152 showed the most significant association with E2 levels measured by GC-MS. This was confirmed in two replication cohorts. Rs2470152 was also significantly associated with E1 levels in all three cohorts (n = 5531) (5).

Meta-analyses of genome-wide association studies (GWASs) enable a comprehensive analysis of the whole genome in a large number of subjects. Chen and colleagues performed a GWAS in 3495 Chinese men in which E2 concentrations were determined using an immunoassay. They found two independent SNPs in the *CYP19A1* gene to be associated with E2 levels (rs2414095 and rs2445762) (6). These findings further strengthened the evidence for a major role of *CYP19A1* in the regulation of serum E2 levels in men, but because of the relatively small sample size and low power, genetic loci in other regions of the genome could have been missed. To date, no GWAS has been performed in men of European origin. In women, a smaller GWAS meta-analysis of 1583 postmenopausal women found no genome-wide significant SNPs. Among variants that were suggestively associated with E2, several were located at the *CYP19A1* locus (7).

Both E2 and testosterone regulate bone mass (8). Studies of men with nonfunctional estrogen receptor alpha (9), and inactivating mutations of the *CYP19A1* gene (10), have demonstrated that estrogens are important for peak bone mass acquisition in men. Population-based studies have shown that in men, low serum levels of E2 are associated with a lower bone mineral density (BMD), higher rates of bone loss, and an increased risk of fractures (8, 11–14). Some studies also show a smaller contribution of testosterone to BMD in men (8, 11). The relative contribution of androgens vs estrogens in the regulation of bone mass in men remains incompletely understood, and studies showing evidence of a causal effect of serum E2 on BMD in men are still sparse (15).

Mendelian randomization is a method used to strengthen or refute the causality of a biomarker, such as E2, and an outcome measure of interest, such as BMD, when a randomized controlled trial is not possible. Mendelian randomization uses genetic data and relies on the principle that, because of the random assortment of genetic variants at conception, these genetic variants are independent of many factors that bias observational studies, such as confounding and reverse causation. Therefore, if a biomarker is etiologically involved in an outcome measure, the genetic factors that influence the biomarker will also influence the outcome measure (16). To date, no Mendelian randomization has been performed to investigate causality between E2 levels and BMD in men.

Case reports of men with aromatase deficiency from an inactivating mutation of the *CYP19A1* gene, mechanistic animal studies and clinical studies also suggest that estrogen signaling through estrogen receptor alpha is important for insulin sensitivity in men (17–23). Thus, genetic factors regulating estrogen levels may also be of relevance for the regulation of insulin sensitivity in men.

Here, we present the results of a GWAS of estrogen levels combining several population-based cohorts of men of European origin. We also present results of our analyses of the association of resultant genome-wide significant associations with two major estrogen related traits: BMD and insulin sensitivity.

## Methods

### Study samples

The discovery stage of the E2 GWAS included 11,097 men of European origin drawn from nine epidemiological cohorts: the Framingham Heart Study (FHS), the Gothenburg Osteoporosis and Obesity Determinants (GOOD) study, the Invecchiare in Chianti study, the Ludwigshafen Risk and Cardiovascular Health (LURIC) study, the Multi-Ethnic Study of Atherosclerosis study, the Osteoporotic Fractures in Men (MrOS) Sweden Gothenburg study, the MrOS Sweden Malmö study, the MrOS US study, and the Rotterdam 1 study (RS1). Replication of one SNP displaying considerable heterogeneity in genome-wide significant fixed-effect models but nominal significance only in random effects models, was performed in the European Male Ageing Study (EMAS; n = 1641). EMAS is a cohort of men predominantly of European origin, with only 0.62% (n = 21) of the sample used here being of non-European descent.

The discovery stage of the E1 GWAS included 7570 men of European origin drawn from six of the previously mentioned cohorts: FHS, GOOD, MrOS Sweden Gothenburg, MrOS Sweden Malmö, MrOS United States, and RS1.

Exclusion criteria included chemical or surgical castration and/or medications affecting sex hormones such as steroid 5-alpha reductase inhibitors and sex hormone antagonists. All studies were approved by local ethics committees and all participants provided written informed consent. Characteristics of the study samples and detailed descriptions of the participating cohorts, genotyping, quality control, and imputation procedures are provided in the Supplemental Appendix and Supplemental Tables 1, 2, and 3.

### Sex hormone measurements

In six discovery cohorts (FHS, GOOD, MrOS Sweden Gothenburg, MrOS Sweden Malmö, and MrOs United States), measurements of E1 and E2 were performed using either GC-MS or liquid chromatography tandem mass spectrometry. In the remaining discovery cohorts (LURIC, Invecchiare in Chianti, Multi-Ethnic Study of Atherosclerosis, and RS1) measurements were performed using immunoassays. In the replication cohort (EMAS), E2 was measured using the GC-MS technique. Methods for all measurements are given in the Supplemental Appendix.

### Genotyping and statistical analyses

Nine discovery and one replication study populations were genotyped using a variety of genotyping platforms including Illumina (HumanHap 550k, 610k, 1M-Duo, Omni1-Quad, Omni express) and Affymetrix (500K Dual GeneChip + 50K gene-centered MIP set, Array 6.0) (Supplemental Table 2). To increase genomic coverage and allow the evaluation of the same SNPs across as many study populations as possible, each study imputed genotype data based on the HapMap CEU Build 36. Algorithms were used to infer unobserved genotypes in a probabilistic manner using either MACH (http://www.sph.umich.edu/csg/abecasis/MA​CH) or IMPUTE2 (24). We analyzed only those SNPs (genotyped or imputed) that had a minor allele frequency of >0.01 and an imputation quality of ≥0.3. The X chromosome was available for analysis in six cohorts (FHS, GOOD, LURIC, MrOS Sweden Gothenburg, MrOS Sweden Malmö, and MrOS United States) in this study. Imputations of the X chromosome were performed in all of these cohorts except MrOS United States.

Altogether, ∼2.5 million SNPs were tested for association with serum E2 and E1 in the discovery stage. GWAS analyses were performed using an additive genetic linear regression model adjusted for: 1) age and body mass index (BMI; E2 and E1) or 2) age, BMI, testosterone, and sex hormone binding globulin (SHBG; E2 only), in each of the discovery cohorts. In FHS, a linear mixed-effect model with a random effect to account for relationships was used. Imputed genotypes were analyzed in all cohorts, taking the genotype uncertainties into account. The meta-analyses were performed in the METAL software (https://www.sph.umich.edu/csg/abecasis/MACH), using an inverse-variance weighted fixed effect model. Random effects models were used when fixed effect models displayed heterogeneity defined as an *I*^2^ value >50% (25). These models were calculated using the *R* package (http://www.r-project.org). A threshold of *P* < 5×10^−8^ was established *a priori* as the level for genome-wide significance in the discovery analyses (26).

Approximate conditional analyses for E2 and E1 were performed using the Genome-wide Complex Trait Analysis (GCTA) software (27), and the genotypes of the European Prospective Investigation of Cancer Norfolk study cohort used as a reference panel to estimate patterns of linkage disequilibrium (LD) (28). The gas chromatography–corrected and quality control–filtered meta-analysis results and a condition list containing the lead SNPs of the final loci were used as input for the conditional analysis. An additional association was declared when the conditional *P* value was below the genome-wide significance threshold. Subsequently, this SNP was added to the list of conditional analysis SNPs and the conditional analysis was performed again in a stepwise fashion until no additional substantial independent associations were found.

### Gene expression analyses

We analyzed associations between identified SNPs associated with serum estrogen levels and gene expression in the eQTL dataset generated by the GTEx Consortium (version 6p), which was obtained from http://www.gtexportal.org/ (29).

### Associations with testosterone

Associations with serum testosterone concentrations were retrieved from the discovery dataset of our previously published GWAS of testosterone levels (30).

### Associations with other traits

We hypothesized, based on data in the literature, that our genome-wide significant SNPs and secondary signals from conditional analyses could be associated with BMD and/or insulin sensitivity. To test these hypotheses, we searched publicly available databases for associations with lumbar spine (LS) and femoral neck (FN) BMD in men [Genetic Factors in Osteoporosis Consortium (GEFOS); www.gefos.org] (31). Data on glycemic traits in men and women combined were downloaded from http://www.magicinvestigators.org/downloads/ (32, 33). Data on glycemic traits in men and women separately were contributed by Meta-Analysis of Glucose and Insulin-Related Traits Consortium (MAGIC) investigators (32, 33). Homeostatic model assessment-estimated insulin resistance was calculated as (fasting insulin × fasting glucose)/22.5.

### Mendelian randomization of serum E2 on BMD

To investigate if E2 has a causal effect on BMD, we performed a summary statistic two sample inverse variance-weighted Mendelian randomization (34). We selected the five top loci from our E2 meta-analysis and extracted summary statistics [*β* and standard error (SE)] from the corresponding SNPs in both our E2 study and the GEFOS study on LS and FN BMD. The variant specific associations were used to create an inverse variance weighted estimate of the causal effect size and its SE.

## Results

We performed a GWAS of serum E2 and E1 concentrations, investigating ∼2.5 million SNPs in up to 11,097 men. In analyses of autosomal chromosomes, all nine discovery cohorts (n = 11,097) were included in the discovery analyses of E2; six cohorts (n = 7570) were included in the discovery analyses of E1.

In analyses of the X chromosome, six cohorts (n = 8953) were included in the discovery analyses of E2 and five cohorts (n = 6917) were included in the discovery analyses of E1.

### E2

**Figure 1.**
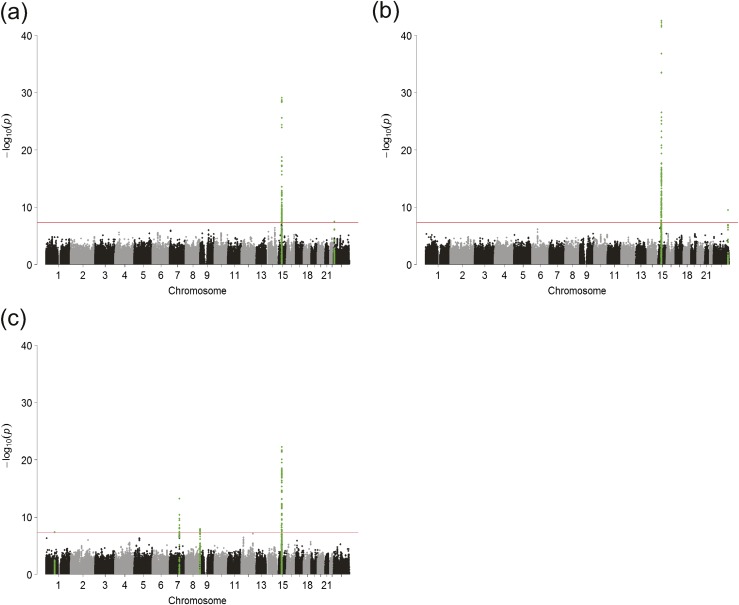
Manhattan plots for the genome-wide meta-analysis results. (a) E2 adjusted for age and BMI; (b) E2 adjusted for, age, BMI, testosterone, and SHBG; and (c) E1 adjusted for age and BMI. Red line indicates *P* = 5 × 10^−8^. Genome-wide significant loci are indicated by green. In the analysis of E1, one SNP on chromosome 1 reached the threshold for genome-wide significance (*P* < 5 × 10^−8^), but had a minor allele frequency of <0.01 in all but two cohorts; therefore, this SNP was discarded from further analyses.

In the model adjusted for age and BMI (model 1), two loci were associated with E2 concentrations at the genome-wide significance threshold of *P* < 5 × 10^−8^ in the discovery analyses [Supplemental Fig. 1(A)]. The strongest association was found within the *CYP19A1* locus on chromosome 15q21.1 (rs727479, effect size 1.39 pg/mL per effect allele; SE, 0.12; *P* = 8.2 × 10^−30^) [Table 1; Fig. 1(a); Supplemental Figs. 2(A) and 3(A)]. This SNP, which is located in the second intron of the gene, showed heterogeneity of effect size across studies as indicated by an *I*^2^ value of 57% (25). To take this heterogeneity into account, we additionally calculated a random effects model, which was also genome-wide significant (effect size = 1.35 pg/mL; SE, 0.19; *P* = 2.0 × 10^−12^).

**Table 1.** SNPs Associated With Serum E2 and E1 Concentrations: Genome-Wide Results of Meta-Analysis

	SNP	Chr	Gene	Location	EA	Freq	Effect	SE	*P*	N
**E2**										
* *Model 1	rs727479	15	*CYP19A1*	51242350	A	0.63	1.39	0.12	8.2 × 10^−30^	11,097
	rs5934505	X	*FAM9B*	8945785	C	0.26	0.67	0.12	3.4 v 10^−8^	8,953
* *Model 2	rs727479	15	*CYP19A1*	51242350	A	0.64	1.42	0.10	3.1 × 10^−43^	10,816
	rs2899472*^a^*	15	*CYP19A1*	51223858	A	0.25	1.13	0.12	1.1 × 10^−8^*^b^*	10,816
	rs16964258*^a^*	15	*CYP19A1*	51313211	G	0.05	2.13	0.25	8.2 × 10^−15^*^b^*	10,816
	rs5951794	X	*MIR*	147350670	G	0.34	0.68	0.11	3.1 × 10^−10^	7,794
**E1**										
* *Model 1	rs2899472	15	*CYP19A1*	51223858	A	0.25	2.41	0.24	5.5 × 10^−23^	7,570
	rs727479*^a^*	15	*CYP19A1*	51242350	A	0.65	2.09	0.22	3.5 × 10^−10^*^b^*	7,570
	rs17277546	7	*TRIM4*	99891948	G	0.95	3.59	0.48	5.8 × 10^−14^	7,570
	rs10093796	8	*CYP11B1/B2*	142897008	T	0.43	1.17	0.20	1.2 × 10^−8^	7,570

Effect size is given per effect allele as picogram per milliliter. Location is given according to human GRCh38/hg38. All cohorts (n = 11,097) were included in the E2 GWAS of chromosomes 1 through 22. The E1 GWAS of chromosomes 1 through 22 included FHS, GOOD, MrOS Sweden Gothenburg, MrOS Sweden Malmö, MrOS United States, and RS1. X chromosome data were available for FHS, GOOD, LURIC, MrOS Sweden Gothenburg, MrOS Sweden Malmö, and MrOS United States. Model 1 is adjusted for age and BMI; model 2 is adjusted for age, BMI, testosterone, and SHBG. Abbreviations: Chr, chromosome; EA, effect allele (*i.e.,* the allele associated with increased serum E2); freq., frequency of effect allele.

^a^ Secondary signal from GCTA analysis.

^b^ Conditional *P* value from GCTA analysis. Fixed-effect meta-analysis *P* values for secondary signals from GCTA analysis: rs2899472 (E2, model 2): *P* = 4.3 × 10^−21^; rs16964258 (E2, model 2): *P* = 2.3 × 10^−17^; rs727479 (E1, model 1): *P* = 2.1 × 10^−22^.

The second locus was found on the X chromosome where one SNP, rs5934505, reached genome-wide significance (*P* = 3.4 × 10^−8^). This SNP is located 79 kb downstream of the *FAMily with sequence similarity 9, member B* (*FAM9B*) gene (Xp22.31) [Table 1; Fig. 1(a); Supplemental Figs. 2(B) and 3(B)]. There was heterogeneity of effect size across studies for this SNP (*I*^2^ = 72%). A random effects model displayed nominal, but not genome-wide, significance in the same direction as the result from the fixed-effect meta-analysis [C-allele associated with higher E2 levels, effect size 0.74 pg/mL per effect allele (SE, 0.24), *P* = 0.002]. Therefore, we attempted replication for rs5934505 in the EMAS cohort (n = 1641). In this cohort, the C-allele was also associated with higher E2 levels; effect size of 1.59 pg/mL per effect allele (SE, 0.39), *P* = 5.2 × 10^−5^.

In the model that was adjusted for testosterone and SHBG levels, in addition to age and BMI [model 2; Supplemental Fig. 1(B)], the associations between E2 and the *CYP19A1* locus remained significant [rs727479: *P* = 3.1 × 10^−43^; Table 1; Fig. 1(b); Supplemental Figs. 2(C) and 3(C)]. In this analysis, the *I*^2^ value was 69%, but the random effects model was genome-wide significant [effect size, 1.42 pg/mL per effect allele (SE, 0.20), *P* = 3.5 × 10^−13^]. A genome-wide significant locus on the X chromosome also appeared in this analysis. rs5951794 (*P* = 3.1 × 10^−10^, *I*^2^ = 6%) is located in the distal part of the long arm on chromosome X (Xq27.3), ∼137 Mb from the *FAM9B* SNP rs5934505 [Table 1; Fig. 1(b); Supplemental Figs. 2(D) and 3(D)].

To identify multiple statistically independent SNPs within the same genomic region, we performed stepwise approximate conditional analyses (GCTA) for each of the genome-wide significant loci. In the model adjusted for testosterone and SHBG, the analysis revealed two additional genome-wide significant SNPs in the *CYP19A1* locus: rs2899472 in intron 4 (conditional *P* = 1.1 × 10^−8^) and rs16964258 in intron 1 (conditional *P* = 8.2 × 10^−15^) [Table 1; Fig. 1(b); Supplemental Figs. 2(C), 3(E), and 3(F)]. In the model adjusted for age and BMI only, no additional independent associations were found.

In model 1, rs727479 explained 0.9% of the overall variance of E2 levels. When the other identified SNP from model 1, rs5934505 (*FAM9B*), was added, 1.1% of the overall variance in E2 levels was explained. In model 2, independent *CYP19A1* SNPs explained 1.3% of the overall variance in E2 levels. When the other genome-wide significant SNP from model 2, rs5951794 (*Chr X*), was added, 1.4% of the overall variance in E2 levels was explained.

### E1

Three genome-wide significant loci, located on chromosomes 7, 8, and 15, respectively, were associated with E1 levels [Supplemental Fig. 1(C)]. The strongest association was found for the *CYP19A1* locus on chromosome 15. The lead SNP was rs2899472 (*P* = 5.5 × 10^−23^) [Table 1; Fig. 1(c); Supplemental Figs. 2(E) and 3(G)]. Because of heterogeneity in effect size at this variant (*I*^2^ = 59%), a random effects model was run, which was genome-wide significant (effect size, 2.55 pg/mL per effect allele; SE, 0.41, *P* = 4.6 × 10^−10^). In conditional analyses of this locus, the SNP with the most significant association with E2, rs727479, was also genome-wide significantly associated with E1 (conditional *P* = 3.5 × 10^−10^) [Table 1; Fig. 1(c); Supplemental Figs. 2(E) and 3(H)].

On chromosome 7, the SNP most significantly associated with E1 levels was rs17277546 (*P* = 5.8 × 10^−14^), located in the 3′ UTR of the *Tripartite motif containing 4* (*TRIM4*) gene [Table 1; Fig. 1(c); Supplemental Figs. 2(F) and 3(I)]. On chromosome 8, the SNP most significantly associated with E1 levels was rs10093796 (*P* = 1.2 × 10^−8^). This SNP is located between the *CYP11B1* and the *CYP11B2* genes [Table 1; Fig. 1(c); Supplemental Figs. 2(G) and 3(J)]. E1 is not derived from testosterone and not bound to SHBG in the circulation; therefore no analyses of E1 adjusted for these parameters were performed.

Independent *CYP19A1* SNPs explained 1.5% of the overall variance in E1 levels. Rs17277546 (*TRIM4)* and rs10093796 (*CYP11B1*/B2) explained 0.5% and 0.1%, respectively, of the variance. In total, 2.1% of the overall variance in E1 levels was explained by these genome-wide significant SNPs.

### Gene expression analyses

In the GTEx database, two of the *CYP19A1* SNPs were robustly associated with the expression level of *CYP19A1*. The alleles associated with higher E2 levels were associated with higher gene expression levels [rs727479: *β* = 0.23, *P* = 1.9 × 10^−5^ (skin); rs2899472: *β* = 0.20, *P* = 9 × 10^−8^ (whole blood)]. Rs727479 was also associated with the expression level of *signal peptide peptidase like 2A* [*β* = 0.18, *P* = 1.3 × 10^−4^ (transformed fibroblasts)], which is located 442 kB upstream of *CYP19A1*. The E1-associated SNP on chromosome 8, rs10093796, was associated with the expression levels of two adjacent genes in several tissues (*Lys6/neurotoxin1*); pancreas: *β* = 0.68, *P* = 5.6 × 10^−9^; *lymphocyte antigen 6 complex, locus K* skin *β* 0.32, *P* = 2.3 × 10^−7^. Both are located 95 and 168 kB, respectively, upstream of *CYP11B1*. The other SNPs in our study were not associated with expression levels in the GTEx database.

### Associations with estrogen-related traits

To further investigate the physiological relevance of our E2 GWAS findings, we performed look-up analyses of other GWAS that had data on phenotypes known or suspected to be related to E2 levels.

### Testosterone

**Figure 2.**
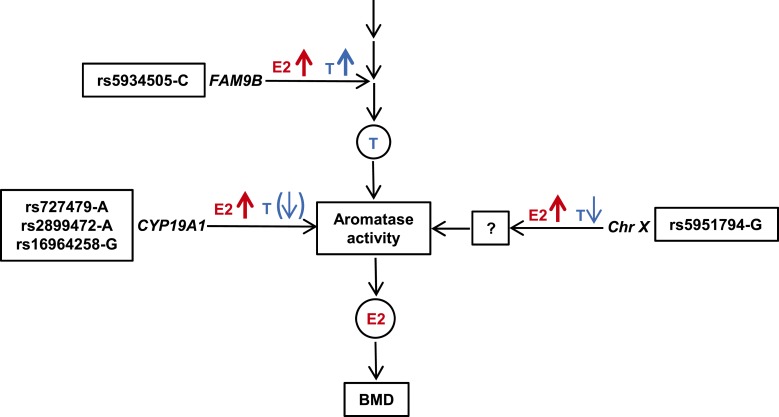
Proposed mechanisms underlying the associations between genome-wide significant SNPs and serum levels of E2 and T. SNPs associated with elevated levels of both E2 and T are expected to be located upstream of T. SNPs associated with elevated levels of E2 but no increase in T levels are expected to affect aromatase activity or E2 clearance. The allele associated with increased serum E2 is given for each SNP. Upwards arrow represents increase, downward arrow represents decrease, and arrow in parentheses represents nonsignificant decrease. The proposed effect of E2 on BMD is also indicated. T, testosterone.

To better understand the mechanism underlying the association between our E2-related SNPs and E2 levels, we studied the association between these SNPs and serum testosterone levels. If the effect of the SNPs on E2 levels was exerted upstream of the aromatase enzyme, one would expect that those SNPs would be associated with higher testosterone as well as higher E2 levels. On the other hand, if the effect of the SNPs on E2 levels were exerted through alteration in either the amount or the activity of the aromatase enzyme, only E2 levels would be expected to be increased, with no increase in testosterone levels. The C-allele of the E2 X chromosome SNP rs5934505 (*FAM9B*) was positively associated with levels of both testosterone and E2, suggesting that the effect of rs5934505 is exerted upstream of aromatase (Table 2; Fig. 2). Indeed, we have previously reported that the X chromosome SNP rs5934505 (*FAM9B*) is associated with circulating testosterone levels in men (*P* = 1.6 × 10^−8^) (30). None of the other E2 SNPs were associated with increased levels of testosterone, suggesting that these SNPs are affecting either the amount or the activity of aromatase or E2 clearance. In fact, the G-allele of the other E2 X chromosome SNP, rs5951794, was associated with increased E2 levels and slightly decreased testosterone levels [effect size, −7.68 ng/dL per effect allele (SE, 3.05), *P* = 0.01] (Table 2; Fig. 2). Additionally, for *CYP19A1* SNPs, there were indications of associations with testosterone in the opposite direction compared with E2, but these associations did not reach statistical significance (rs727479 *P* = 0.05 and rs16964258: *P* = 0.26) (Table 2; Fig. 2).

**Table 2.** Look-Up of Genome-Wide Significant Lead SNPs and Testosterone in Men

Chr	Gene	SNP	EA	Freq	Testosterone, Adjusted for SHBG			
					Effect	SE	*P*	n*^a^*
15	*CYP19*	rs727479	A	0.64	−4.86	2.49	0.051	8366
15	*CYP19*	rs2899472	A	0.26	−0.030	2.82	0.99	8366
15	*CYP19*	rs16964258	G	0.05	−6.85	6.07	0.26	8366
X	*FAM9B*	rs5934505	C	0.26	18.10	3.20	**1.6 × 10^−8^**	4599
X	*MIR*	rs5951794	G	0.34	−7.68	3.05	**1.2 × 10^−2^**	4599

Effect size is given per effect allele as nanogram per deciliter. Numbers in bold represent statistical significance. Testosterone levels were retrieved from our previous GWAS of testosterone levels (30).

^a^ Total number of study participants, information for individual SNPs not available.

### BMD

The primary SNP in *CYP19A1*, rs727479, and the secondary signals rs2899472 and rs16964258, were all significantly associated with LS BMD in men (*P* ≤ 0.01; Table 3). Rs727479 and rs2899472 were also associated with FN BMD in men (*P* < 0.01). The direction of the effect was the same for all markers (*i.e.* alleles associated with higher levels of E2) were associated with a higher BMD. Moreover, rs5934505 (*FAM9B*) was associated with both FN (*P* = 0.01) and LS (*P* = 7 × 10^−6^) BMD. As in the case of *CYP19A1* SNPs, the allele associated with higher E2 levels was associated with a higher BMD (Table 3).

**Table 3.** Look-Up of Genome-Wide Significant Lead SNPs and BMD in Men

					BMD							
					LS				Femoral Neck			
Chr	Gene	SNP	EA	Freq	Effect	SE	*P*	n*^a^*	Effect	SE	*P*	n*^a^*
15	*CYP19*	rs727479	A	0.70	0.068	0.015	**1.1 × 10^−5^**	9980	0.059	0.015	**1.2 × 10^−4^**	9980
15	*CYP19*	rs2899472	A	0.28	0.047	0.017	**7.4 × 10^−3^**	9980	0.052	0.018	**2.4 × 10^−3^**	9980
15	*CYP19*	rs16964258	G	0.06	0.10	0.039	**1.0 × 10^−2^**	9980	0.065	0.029	0.09	9980
X	*FAM9B*	rs5934505	C	0.26	0.059	0.012	**7.2 × 10^−6^**	9980	0.031	0.012	**1.2 × 10^−2^**	9980
X	*MIR*	rs5951794	G	0.34	0.016	0.012	0.19	9980	0.005	0.012	0.67	9980

Effect size for BMD is given as standardized values per copy of the SNP allele from fixed-effects meta-analysis. Numbers in bold represent statistical significance after Bonferroni correction for two phenotypes (LS BMD and FN BMD).

^a^ Total number of study participants, information for individual SNPs not available.

### Mendelian randomization E2 and BMD

**Figure 3.**
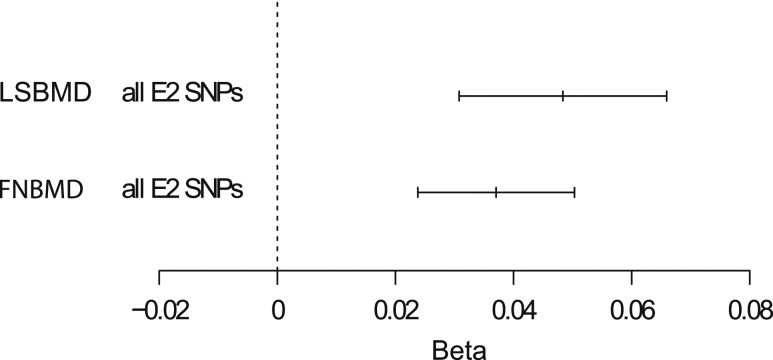
Forest plot of Mendelian randomization analyses showing the effect of E2 on BMD. Effect size of E2 on BMD expressed as SD increase in BMD per pg/mL E2. The horizontal lines represent confidence interval; the central vertical line represents precision. The values are based on a meta-analysis of all five E2-associated SNPs (rs727479, rs2899472, rs16964258, rs5934505, rs5951794). The horizontal axis shows the scale of the effects. FNBMD, femoral neck bone mineral density; LSBMD, lumbar spine bone mineral density.

The data from the GEFOS database show associations between individual SNPs and BMD, but do not provide information on possible causality between the E2 levels resulting from these SNPs and BMD. To overcome this, we performed a summary statistic Mendelian randomization analysis, which suggested that there is a causal effect of serum E2 on BMD. A 1 pg/mL genetically increased E2 was associated with a 0.048 standard deviation (SD) (SE, 0.008), *P* = 2.8 × 10^−12^ increase in LS BMD. For the femoral neck, the increase was 0.037 SD (SE, 0.007, *P* = 4.4 × 10^−8^) (Fig. 3).

### Insulin sensitivity

The publicly available GWAS results for measures of insulin sensitivity included only autosomal chromosomes, and did not include results for men and women separately. Thus the following results apply for men and women combined. Insulin resistance expressed as homeostatic model assessment-estimated insulin resistance was negatively associated with the E2 increasing A alleles of rs727479 (*P* = 0.004) and rs2899472 (*P* = 0.003) in *CYP19A1*. This was due to a negative association of these alleles with fasting insulin (*P* = 0.003 for rs727479; *P* = 0.017 for rs2899472) (Supplemental Table 4). Adjustments for BMI had no effect on the results (BMI-adjusted fasting insulin, *P* = 0.002 for rs727479 and *P* = 0.031 for rs2899472). There were no associations with fasting glucose for these SNPs. The MAGIC investigators also provided us with data not publicly available on fasting insulin and fasting glucose for men and women separately (fasting insulin: men, n ≈ 26,000; women, n ≈ 32,000; fasting glucose: men, n ≈ 36,000; women, n ≈ 43,000). In this dataset, the association between rs727479 and fasting insulin was significant in women [*β* = −0.014 (SE, 0.004), *P* = 0.002]. In men, the direction of the association was the same as in women, but was not statistically significant [rs727479: *β* = −0.006 (SE, 0.005), *P* = 0.19].

## Discussion

In this GWAS, SNPs in the *CYP19A1* gene showed the strongest associations with both E1 and E2 levels. This confirms data from previous studies (5, 6, 35) and establishes *CYP19A1* as an important genetic regulator of estrogen levels in men. We found three independent signals in *CYP19A1*, which extends the results from previous studies. We also identified two additional signals for E2 on chromosome X and two additional signals for E1 on chromosomes 7 and 8, respectively. Moreover, SNPs found to be associated with E2 levels in this study were also associated with known or suspected estrogen-related traits including BMD and insulin sensitivity. Mendelian randomization analysis using the independent E2 SNPs suggests a causal effect of E2 on BMD in men.

The finding of several independent signals for both E1 and E2 in *CYP19A1* is consistent with the findings in the previously reported GWAS in Chinese men, where two independent SNPs were found. This strengthens the conception that the regulation of estrogen levels is governed by more than one signal in the gene. The organization of *CYP19A1* is rather complex. The gene consists of a 30-kb coding region and a 93-kb regulatory region including 10 tissue-specific promoters (36). There are four blocks of LD in the gene. Rs727479, which displayed the most important association with E2 levels in our study, is located in intron 2 in LD block 4, which covers 50 kB, including the entire coding region, exons/promoters I.6, I.3, and PII, through 5.8 kb downstream of exon 10 (37). Rs727479 has been associated with E2 levels in previous candidate gene studies investigating haplotype-tagging SNPs in *CYP19A1* as well as in more comprehensive studies investigating larger numbers of SNPs in many genes in both men (35, 38, 39) and postmenopausal women (40). Moreover, rs727479 was the second-most significant SNP in the GWAS of E2 levels in postmenopausal women performed by Prescott and colleagues, although it did not reach genome-wide significance (*P* = 5 × 10^−7^), perhaps because of the relatively low number of study participants (7). In all of these studies, the direction of the effect was the same as in our study: the A allele was associated with higher E2 levels. The most significant SNP in the male Chinese GWAS performed by Chen and colleagues, rs2414095, is in very strong linkage (*r*^2^ = 0.96) with rs727479 (6); it is also located in intron 3 in LD block 4. The findings from our gene expression analyses that rs727479 is associated with the expression of *CYP19A1* in two tissues further support the relevance of this SNP in the regulation of E2 levels.

To our knowledge, the *CYP19A1* loci rs2899472 and rs16964258 have not been linked to E2 levels in previous studies. Rs2899472 is located in intron 4, in LD block 4. Rs16964258 is located in a different region of the gene; intron 1, between LD blocks 1 and 2. Interestingly, the SNP most significantly associated with estrogen levels in our previous extended candidate gene study, rs2470152 (5), is also located in this region, 10 kb downstream of rs16964258. The D′ for rs2470152 and rs16964258 is 1.0 but the *r*^2^ is 0.062, indicating that the SNPs are probably linked but, because of different allele frequencies, they are not proxy SNPs of one another.

The signal in the *FAM9B* region on the X chromosome, rs5934505, has not been associated with E2 levels before, but associations of this locus with testosterone levels are known from our earlier testosterone GWAS (30), a finding that was later replicated by Jin and colleagues in a smaller GWAS in men (n = 3225) (41). Because testosterone is the precursor of E2, it is likely that the association of rs5934505 in the *FAM9B* region with E2 levels is mediated through the regulation of testosterone production and not through the conversion of testosterone to E2 *per se*. Rs5934505 is located in a CNV-insertion area (Xp22), 145 kb upstream of *FAM9A* and 79 kb downstream of *FAM9B* genes. Both genes are expressed exclusively in the testes and share 46% amino acid identity. Very little is known about their functions (42). The *Kallman syndrome 1* (*KAL1*) gene is located 214 kb downstream of rs5934505. *KAL1* encodes the extracellular matrix glycoprotein anosmin-1 implicated in the embryonic migration of gonadotropin-releasing hormone and olfactory neurons. Deleterious mutations in *KAL1* cause X-linked Kallmann syndrome, characterized by hypogonadotropic hypogonadism and anosmia (43), but there are no previous data supporting that minor alterations in the function of KAL1 are associated with sex steroid levels. Moreover, rs5934505 is correlated (*r*^2^ = 0.35) with another SNP, rs5978985, in this region, which was associated with male puberty in a recent GWAS (44).

The other signal on the X chromosome, rs5951794, has not previously been associated with sex steroid levels, and the mechanism underlying the association in our study is not known. In contrast to rs5934505 *(FAM9B)*, rs5951794 was not associated with higher testosterone levels. Therefore, the effect of this SNP would be expected to be exerted through alteration in the amount or activity of the aromatase enzyme or through regulation of E2 clearance. In fact, rs5951794 was associated with slightly lower levels of testosterone. This might be the result of E2-mediated suppression of luteinizing hormone, which in turn would result in decreased testosterone levels. Rs5951794 is located approximately 65 kb downstream of a region rich in micro-RNAs (506 through 510, 513, and 514), expressed mainly in the testes (45). Aside from the micro-RNA cluster, *Fragile X mental retardation 1* is the closest gene located approximately 700 kb downstream of rs5951794. Keeping the distance in mind, one could speculate that rs5951794 could affect the regulation of *Fragile X mental retardation 1*, a gene that, in addition to its crucial role in the pathogenesis of fragile X syndrome–associated mental retardation, is also the leading molecular cause of premature ovarian failure (46).

The E1 signal rs17277546 in the *TRIM4* gene has also been shown to be associated in our previous GWAS of dehydroepiandrosterone sulfate (DHEAS) concentrations (47). Serum levels of DHEAS and dehydroepiandrosterone are highly collinear (48). Serum levels of DHEAS could therefore be a marker of serum levels of DHEA. In our earlier GWAS, the G allele was associated with higher levels of DHEAS; in the current study, the G allele was associated with higher levels of E1. Thus, an increased amount of adrenal-derived precursors for estrogen synthesis is a possible explanation for the present findings. *TRIM4* is a member of the *TRIM* family. Members of this family have been implicated in many biological processes, including cell differentiation, apoptosis, and transcriptional regulation (49). The mechanism relating rs17277546 to DHEAS levels is not known, but in our previous GWAS, we found that rs17277546 is strongly associated with expression levels of *TRIM4* in cell lines from liver and adipose tissue in publicly available databases. This indicates that rs17277546 is a functional SNP or is linked to such an SNP (47).

The chromosome 8 signal, rs10093618, is located 1.5 kb upstream of the *CYP11B1* gene. The product of *CYP11B1*, the steroid 11*β*-hydroxylase enzyme, catalyzes the conversion of 11-deoxycortisol to cortisol, representing the final step in cortisol biosynthesis, and 11-deoxycorticosterone to corticosterone. Deficiency of this enzyme leads to congenital adrenal hyperplasia. Hyperandrogenism is a hallmark of this condition because accumulated precursors are shunted into the androgen synthesis pathway (50). One could thus speculate that rs10093618, or an unknown variant linked with it, affects the production or efficiency of the steroid 11*β*-hydroxylase enzyme, and thereby regulates the level of adrenal precursors for the sex steroid synthesis pathway, notably androstenedione, which is a direct precursor in E1 biosynthesis.

Because serum E2 levels in men are positively associated with BMD, the SNPs associated with higher E2 levels would be expected to be associated with higher BMD. In fact, in our previous extended candidate gene study, there was such an association between the lead *CYP19A1* SNP, rs2470152, and BMD (5). Thus, the association in the current study between E2-associated SNPs in *CYP19A1* as well as *FAM9B* and BMD is a plausible finding. In fact, rs5934505 is in complete linkage (*r*^2^ = 1.0) with rs5934507, which was identified as the only male-specific signal in our previous GWAS of BMD (31). Because of the known association of rs5934505 with testosterone, the BMD signal was thought to be mediated via testosterone levels in the BMD GWAS. Given the findings in the current study of an association between rs5934505 and E2, it seems more likely that the association with BMD is mediated at least in part via E2 levels rather than solely via a direct effect of testosterone (Fig. 3).

Although an association between serum E2 levels and BMD in men has been shown in earlier association studies, a causal relation has not been demonstrated. In this study, using Mendelian randomization analysis, we provide evidence that there is a causal effect of E2 on BMD. For instance, in the RS1 cohort, where the E2 levels were 12.7 pg/mL (SD, 6.6), 1 SD of genetically instrumented decrease in E2 would result in a 6.6 × 0.048 = 0.32 SD decrease in LS BMD and 6.6 × 0.037 = 0.24 SD decrease in FN BMD.

According to Johnell and coworkers, the relative risk for hip fracture in men aged 65 years was 2.94 (95% confidence interval, 2.02 to 4.27) for each SD decrease in FN BMD (51). Using this information of the association between FN-BMD and hip fracture risk together with the causal effect of serum E2 on FN-BMD as estimated in the present Mendelian randomization analysis, 1 SD (using the SD of serum E2 from the RS1 cohort) decrease in genetically instrumented E2 level could increase the relative risk for hip fracture by 47%.

In this study, SNPs in *CYP19A1* that were associated with higher E2 levels were also associated with improved insulin sensitivity and lower fasting insulin in men and women combined. In men, the role of estrogens in the regulation of insulin sensitivity is not fully understood. However, mechanistic studies and clinical trials suggest that estrogen signaling is important in the regulation of insulin sensitivity in men (18, 20, 22, 23). Furthermore, men with aromatase deficiency resulting from an inactivating mutation of the *CYP19A1* gene are overweight or obese, and display and insulin resistance, which often improves with estrogen replacement therapy (17).

The strengths of our study include the large sample size, with 11,097 men in the discovery analysis of E2 levels, and the large proportion of serum samples analyzed using the MS technique. This enabled us to find multiple signals in the *CYP19A1* locus and signals on other chromosomes for both E1 and E2. A potential weakness of our study is that not all samples were analyzed by MS. As a result of the lower specificity of the immunoassays, weaker genetic signals might have been missed. It is likely that future studies with even larger numbers of samples analyzed by MS could uncover signals not found in this study. Nevertheless, we believe that, because of the large proportion of samples analyzed by MS, our findings are robust and the risk for false-positive signals is low. We also found SNP associations with BMD and measures of insulin sensitivity. Additionally, the Mendelian randomization analysis provides evidence of a causal effect of E2 on BMD in men. The mechanisms underlying some of the associations in our study should be further investigated to expand our understanding of the regulation of sex steroid levels.

## Supplementary Material

Supplemental Materials

## Abbreviations:

BMD: bone mineral density
BMI: body mass index
DHEAS: dehydroepiandrosterone sulfate
E1: estrone
E2: estradiol
EMAS: European Male Ageing Study
*FAM9B*: *FAMily with sequence similarity 9, member B*
FHS: Framingham Heart Study
FN: femoral neck
GC-MS: gas chromatography-mass spectrometry
GEFOS: Genetic Factors in Osteoporosis Consortium
GOOD: Gothenburg Osteoporosis and Obesity Determinants
GWAS: genome-wide association study
*KAL1*: *Kallman syndrome 1*
LD: linkage disequilibrium
LS: lumbar spine
LURIC: Ludwigshafen Risk and Cardiovascular Health
MrOS: Osteoporotic Fractures in Men
RS1: Rotterdam 1 study
SD: standard deviation
SE: standard error
SHBG: sex hormone binding globulin
SNP: single nucleotide polymorphism
*TRIM4*: *Tripartite motif containing 4.*
