# Supplementary material for: Genetic Determinants of Circulating Estrogen Levels and Evidence of a Causal Effect of Estradiol on Bone Density in Men
Source: J Clin Endocrinol Metab. 2018 Jan 9;103(3):991–1004. doi: 10.1210/jc.2017-02060 (PMC5868407; doi:10.1210/jc.2017-02060)
Supplement: Supplemental Materials [file jc.2017-02060_supplemental_figure_1.pdf]

Supplemental Figures 1A-C. Quantile-quantile plots of the genome-wide association results of the inverse-variance weighted meta-analysis.

(A)

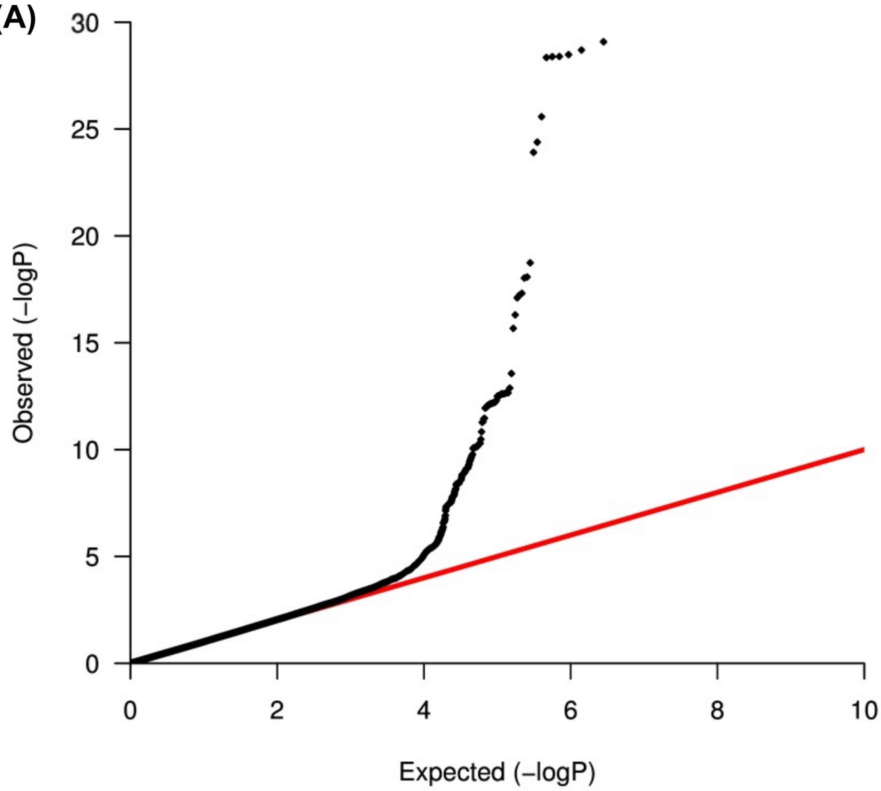

(B)

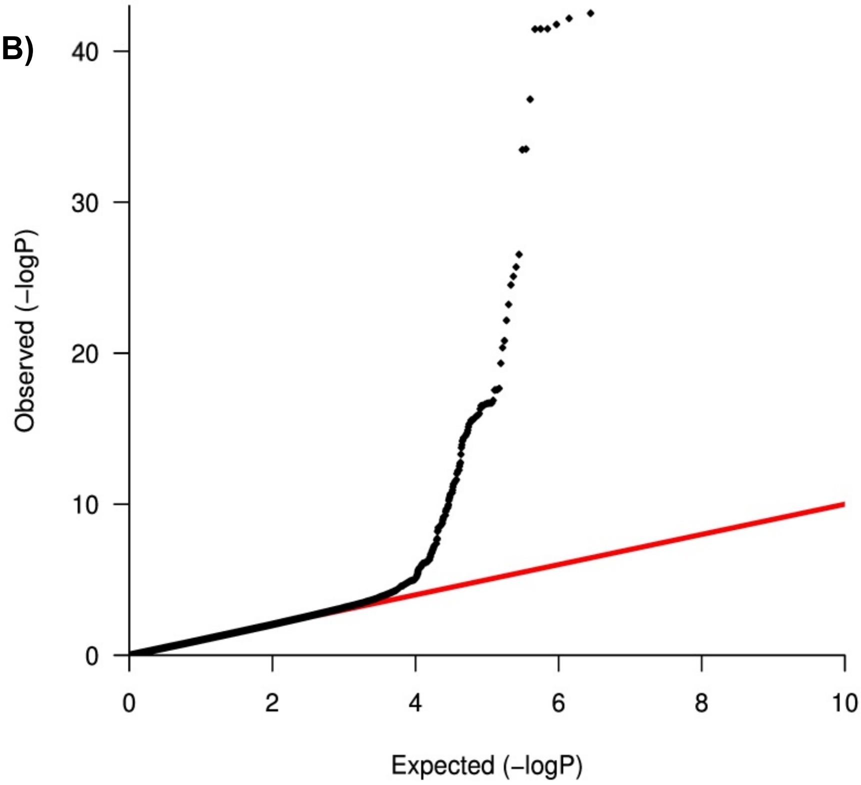

(C)

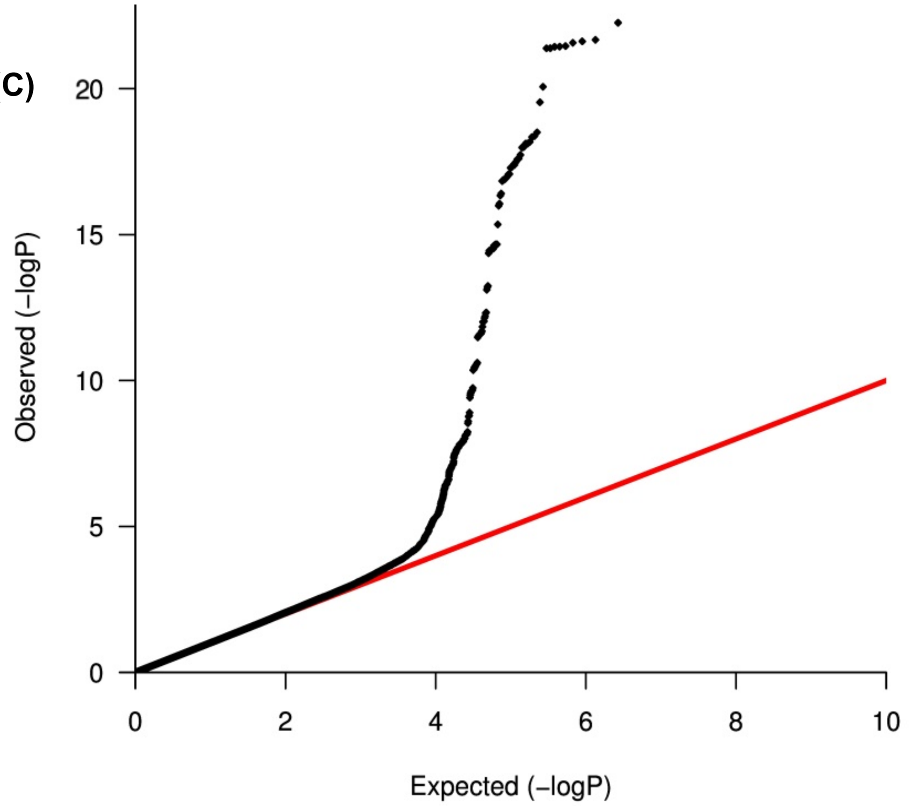

(A) E2 adjusted for age and BMI, (B) E2 adjusted for age, BMI, testosterone and SHBG, and (C) E1 adjusted for age and BMI.

Supplemental figures 2A-G. Regional association plots for single-nucleotide polymorphisms associated with E2 or E1

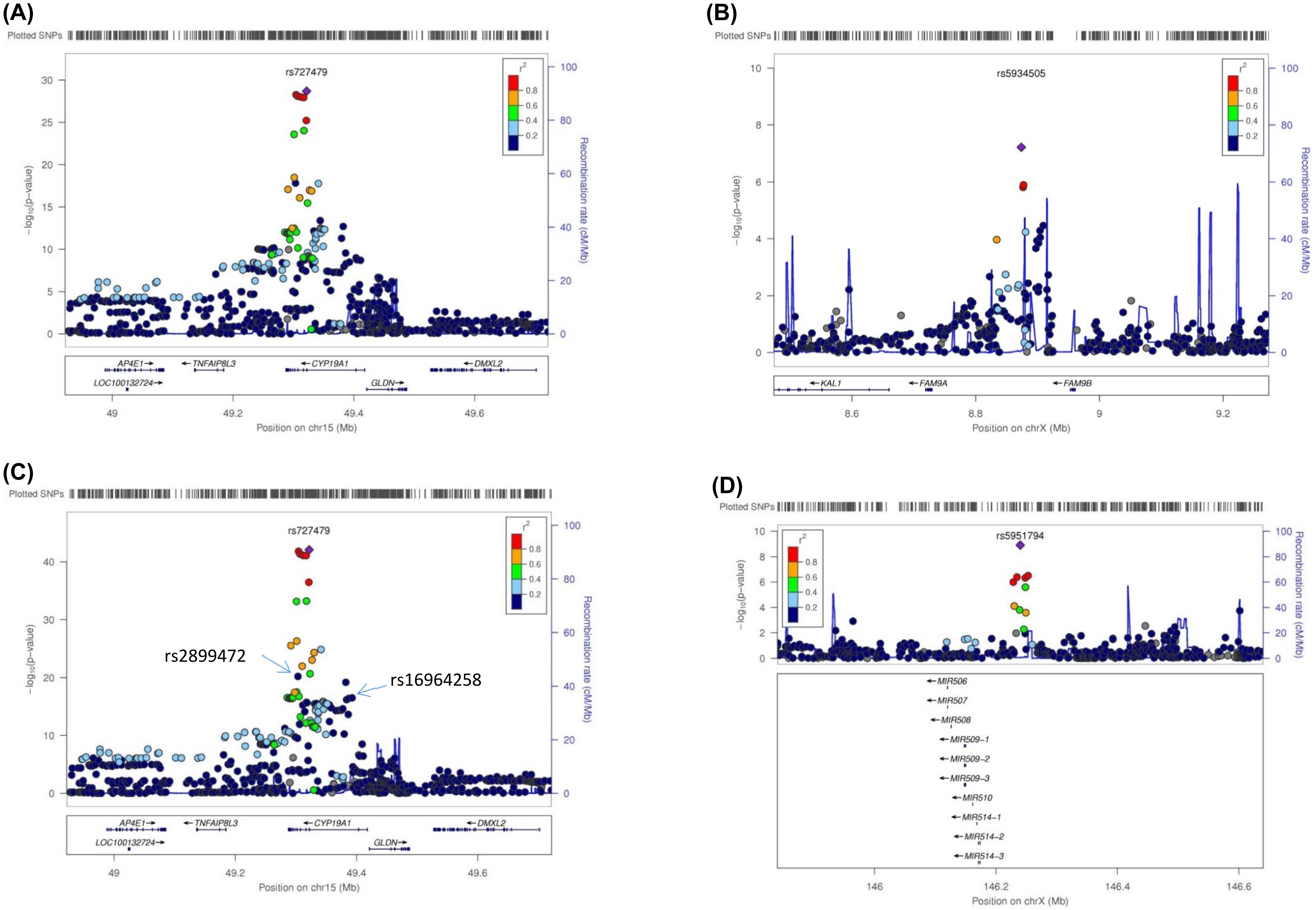

(E)

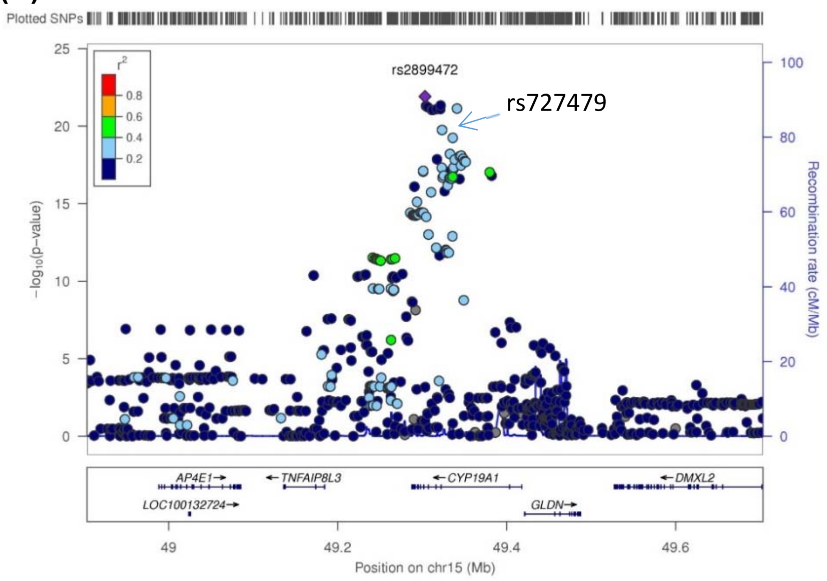

Plotted SNPs
rs2899472
rs727479
r²
−log₁₀(p−value)
Recombination rate (cM/Mb)
AP4E1→
←TNFAIP8L3
←CYP19A1
←DMXL2
LOC100132724→
GLDN→
Position on chr15 (Mb)

(F)

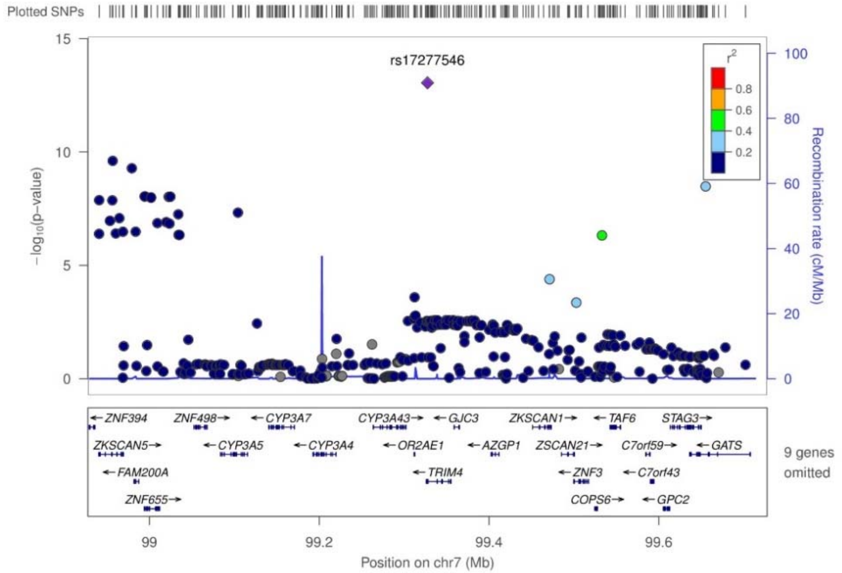

Plotted SNPs
rs17277546
r²
−log₁₀(p−value)
Recombination rate (cM/Mb)
←ZNF394
ZNF498→
←CYP3A7
CYP3A43→
←GJC3
ZKSCAN1→
←TAF6
STAG3→
ZKSCAN5→
←CYP3A5
←CYP3A4
←OR2AE1
←AZGP1
ZSCAN21→
C7orf59→
←GATS
←FAM200A
←TRIM4
←ZNF3
←C7orf43
ZNF655→
COPS6→
←GPC2
9 genes omitted
Position on chr7 (Mb)

(G)

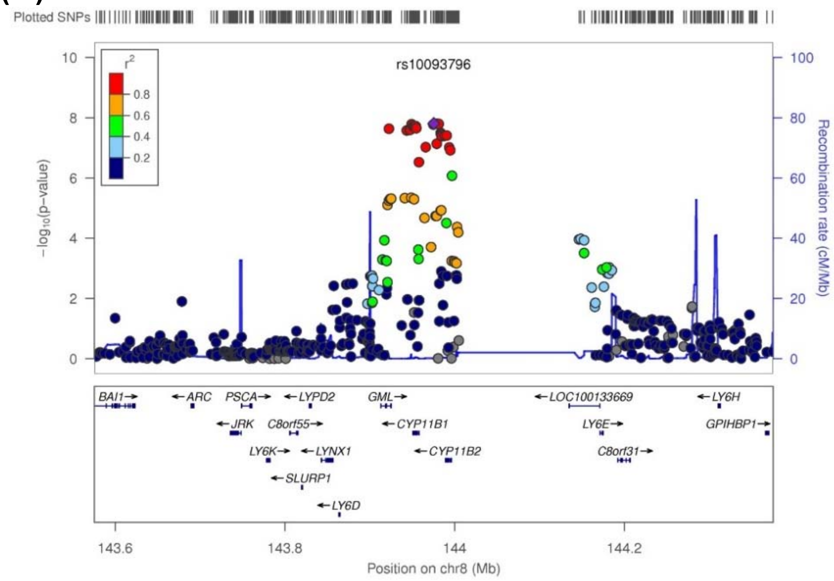

Plotted SNPs
rs10093796
r²
−log₁₀(p−value)
Recombination rate (cM/Mb)
BAI1→
←ARC
PSCA→
←LYPD2
GML→
←LOC100133669
←LY6H
←JRK
C8orf55→
←CYP11B1
LY6E→
GPIHBP1→
LY6K→
←LYNX1
←CYP11B2
C8orf31→
←SLURP1
←LY6D
Position on chr8 (Mb)

(A) rs727479, E2 adjusted for age and BMI, (B) rs5934505, E2 adjusted for age and BMI, (C) rs727479, rs2899472 and rs16964258, E2 adjusted for age, BMI, testosterone and SHBG, (D) rs5951794, E2 adjusted for age, BMI, testosterone and SHBG, (E) rs2899472 and rs727479, E1 adjusted for age and BMI, (F) rs17277546, E1 adjusted for age and BMI, (G) rs10093796, E1 adjusted for age and BMI. Location is given according to Human NCBI36/hg18. Independent signals are indicated by purple diamond to evaluate linkage with other single-nucleotide polymorphisms in the region. The r^2 is based on the CEU HapMap II samples. The blue line and right hand Y axis represent CEU HapMap II based recombination rates. The location of secondary signals is marked with arrows in figures C (rs2899472 and rs16964258) and E (rs727479).

Supplemental figures A-J. Forest plots for the top hits from the GWAS.

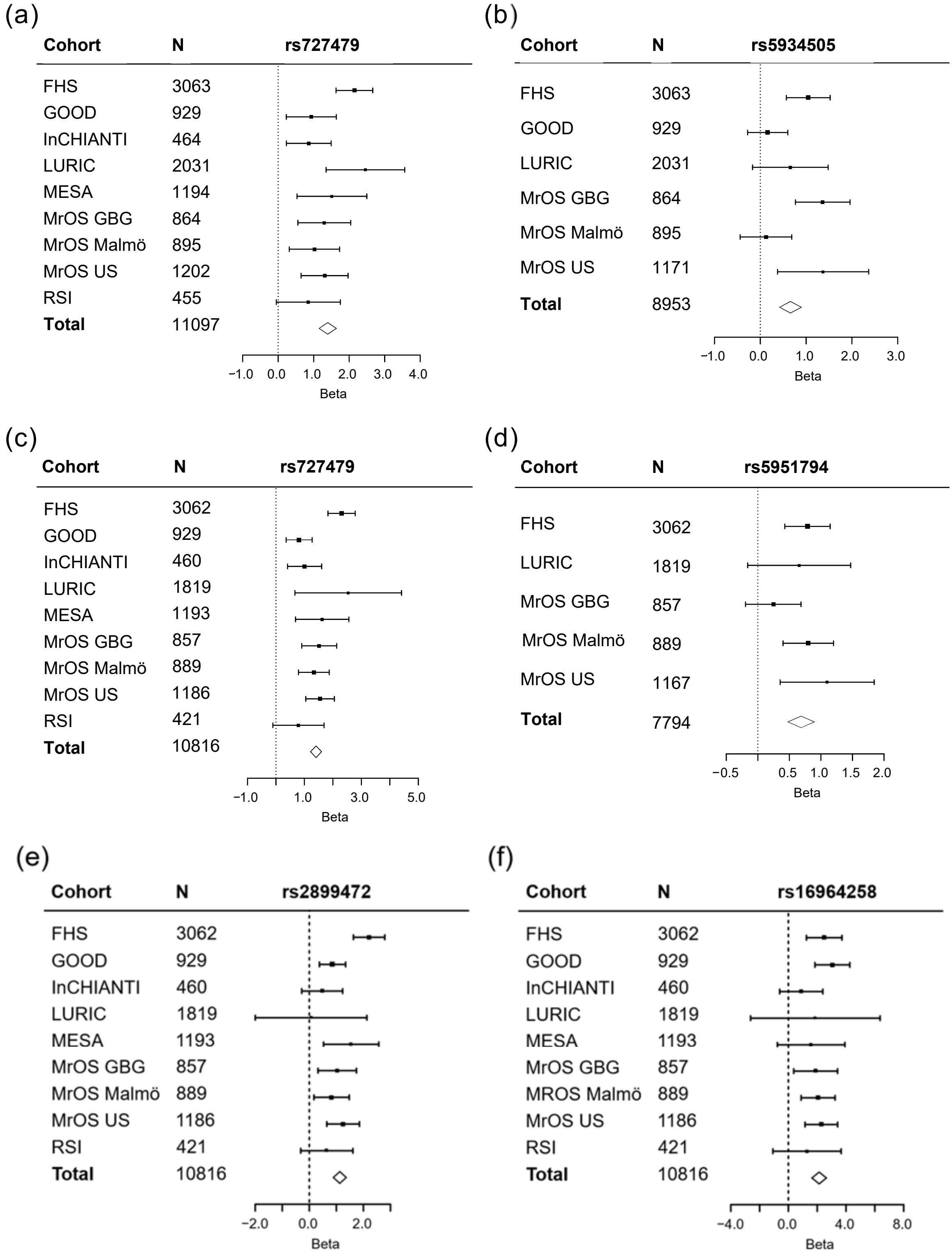

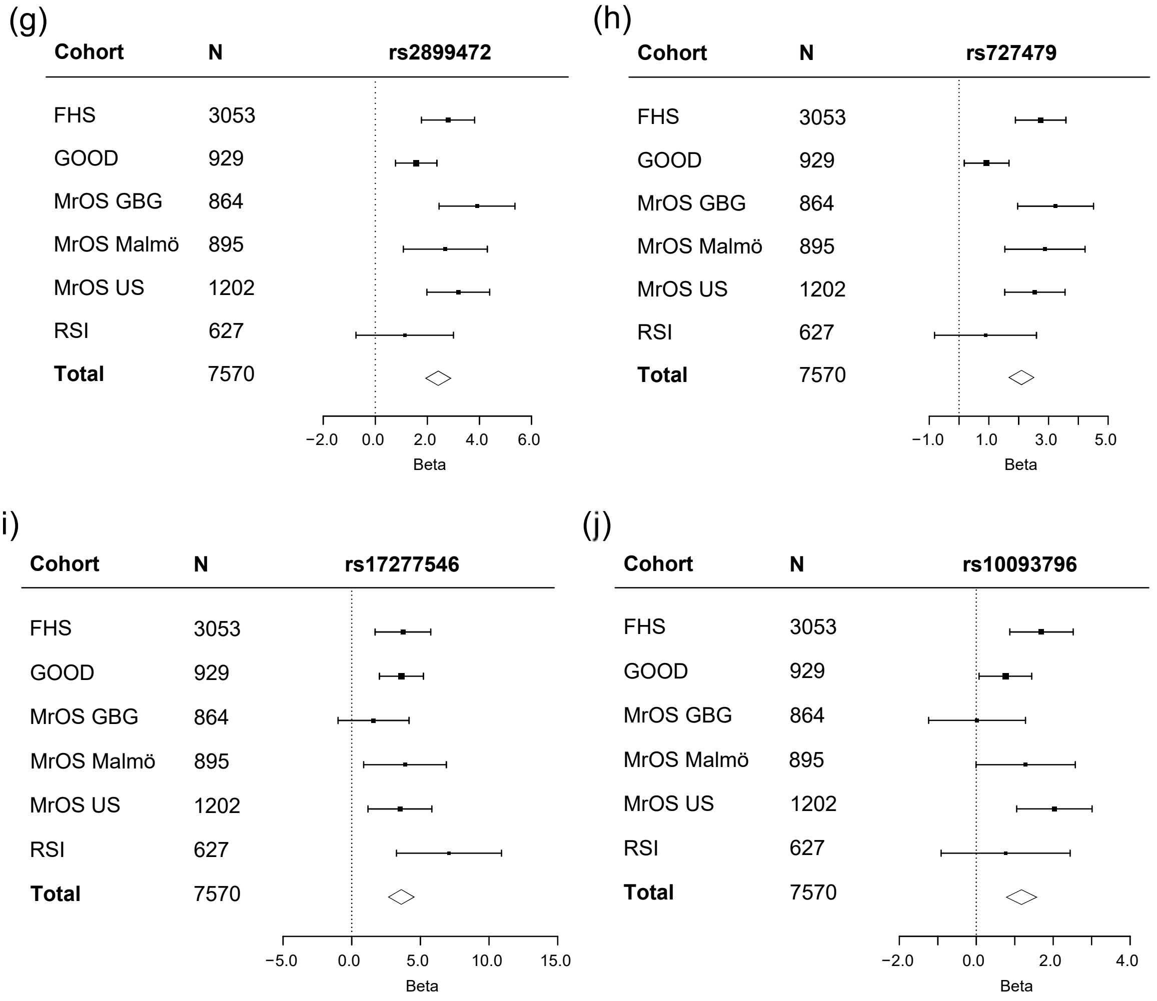

(A) rs727479, E2 adjusted for age and BMI, (B) rs5934505, E2 adjusted for age and BMI, (C) rs727479, E2 adjusted for age, BMI, testosterone and SHBG, (D) rs5951794, E2 adjusted for age, BMI, testosterone and SHBG, (E) rs2899472, E2 adjusted for age, BMI, testosterone and SHBG, (F) rs16964258, E2 adjusted for age, BMI, testosterone and SHBG, (G) rs2899472, E1 adjusted for age and BMI, (H) rs727479, E1 adjusted for age and BMI, (I) rs17277546, E1 adjusted for age and BMI, (J) rs10093796, E1 adjusted for age and BMI. Beta given as pg/ml E2/allele (A-F), or pg/ml E1/allele (G-J). Vertically left, the populations included in the GWAS. The boxes represent precision, and horizontal lines represent the confidence intervals. The diamond represents the pooled effect estimate from the meta-analysis of all cohorts. The horizontal axis shows the scale of the effects.

Supplemental Table 1

Study design, number of individuals, and sample quality control for discovery and replication cohorts

**Study**					**Sample QC**			
**Short name**	**Full name**	**Study design**	**Ethnicity**	**Total sample size**	**Call rate***	**Other exclusions**	**Samples in analyses**	**References**
Discovery Cohorts								
Framingham Study	Framingham Heart Study	Community-based	Caucasian	3318	≥ 97%	1. autosomal heterozygosity < 0.33 or > 0.37<br>2. ethnic outliers (using Eigenstraat)<br>3. missing estradiol or estrone or covariates	3099	**[PMID: 23746309]** Travison TG. et al. The heritability of circulating testosterone, oestradiol, oestrone and sex hormone binding globulin concentrations in men: the Framingham Heart Study, Clin Endocrinol (Oxf). 2014 Feb;80(2):277-82.
GOOD	Gothenburg Osteoporosis and Obesity Determinants study	Population-based	Caucasian	1068	≥ 97.5%	1) heterozygosity > 33%;<br>2) ethnic outliers;<br>3) related individuals and duplicates.	929	**[PMID: 16007330]** Lorentzon, M. et al Free testosterone is a positive whereas free estradiol is a negative predictor of cortical bone size in young Swedish men-The GOOD Study. J Bone Miner Res 20, 1334-1341 (2005).
InCHIANTI Study	InCHIANTI Study	Population-based	Caucasian	1210	≥ 98%	1) heterozygosity > 33%;<br>2) ethnic outliers;<br>3) related individuals and duplicates.	464	**[PMID: 18464913]** Melzer and Perry. et al A genome-wide association study identifies protein quantitative trait loci (pQTLs). PLoS Genetics May 9;4(5) (2008)
LURIC	Ludwigshafen Risk and Cardiovascular Health study	Case-control	Caucasian	3316	>95%	1) related individuals and duplicates<br>2) sex ambiguity<br>3) no available genotypes<br>4) female sex	2031	**[PMID:11258203]** Winkelmann BR. et al. Rationale and design of the LURIC study--a resource for functional genomics,

								pharmacogenomics and long-term prognosis of cardiovascular disease: Pharmacogenomics. 2001 Feb;2(1 Suppl 1):S1-73. Review.
MESA	Multi-Ethnic Study of Atherosclerosis	Population-based	Caucasian	1204	≥ 95%	1) duplicates<br>2) unresolved gender mismatches<br>3) unresolved cryptic duplicates<br>4) missing phenotype	1194	**[PMID: 18849030]** Ouyang P, Vaidya D. et al. Sex hormone levels and subclinical atherosclerosis in postmenopausal women: the Multi-Ethnic Study of Atherosclerosis. Atherosclerosis. 2009 May;204(1):255-61.
MrOS Sweden - Gothenburg	The Osteoporotic Fractures in Men Study - Sweden/Gothenburg	Population-based	Caucasian	1014	≥ 97%	1) excessive autosomal heterozygozity;<br>2) first and second degree relatives;<br>3) genotypic sex mismatch using X and Y chromosome probe intensities and gross chromosome abnormalities	864	**PMID:16598372]** Mellström, D. et al. Free testosterone is an independent predictor of BMD and prevalent fractures in elderly men: MrOS Sweden. J Bone Miner Res 21, 529-535 (2006).
MrOS Sweden - Malmö	The Osteoporotic Fractures in Men Study - Sweden/Malmö	Population-based	Caucasian	1001	≥ 97.5%	1) heterozygosity (F<0.03946);<br>2) ethnic outliers;<br>3) related individuals and duplicates.	895	**[PMID:16598372]** Mellström, D. et al. Free testosterone is an independent predictor of BMD and prevalent fractures in elderly men: MrOS Sweden. J Bone Miner Res 21, 529-535 (2006).
MrOS US	The Osteoporotic Fractures in Men Study - US	Population-based cohort	Caucasian	5994	≥ 97%	1) Reported race/ethnicity other than non-Hispanic white<br>2) Non-consent for genetic studies<br>3) Related individuals<br>4) High BAF variance in more than 5 chromosomes	1202	**[PMID:16084776]** Orwoll E. et al. Design and baseline characteristics of the osteoporotic fractures in men (MrOS) study--a large observational study of the determinants of fracture in older men. Contemporary

								clinical trials. Oct 2005;26(5):569-585. **[PMID:16085466]** Blank JB. et al. Overview of recruitment for the osteoporotic fractures in men study (MrOS). Contemp Clin Trials 26:557–568 (2005)
RS-I	Rotterdam Study	Population-based	Caucasian	5974	≥ 97.5%	1) heterozygosity > 33%;<br>2) ethnic outliers;<br>3) related individuals and duplicates.	455 E2 / 627 E1	**[PMID: 26386597]** Hofman A. et al. The Rotterdam Study: 2016 objectives and design update. Eur J Epidemiol. 2015 Aug;30(8):661-708.
Replication cohort								
EMAS	European Male Ageing Study	Population-based	Caucasian	3369	≥ 97%	No exclusions were made	1641	**[PMID: 18328041]**<br>Lee, DM. et al The European Male Ageing Study (EMAS): design, methods and recruitment. Int J Androl 32:11-24 (2009).

* Sample genotyping success rate; i.e. minimum percentage of successfully genotyped SNPs of GWAs per sample

Supplemental Table 2

Additional genotyping information for the 10 cohorts included in the genome-wide association study meta-analysis

	Genotyping platform	Genotype calling algorithm	Imputation software	SNPs in meta-analysis	Analyses software
**Discovery Cohorts**					
FHS	Affymetrix 500K Dual GeneChip +50K gene-centered MIP set	BRLMM	MACH	2 471285	Kinship R-Package
GOOD	Illumina Infinium HumanHap 610K	BeadStudio	MACH	2 543 887	MACH2QTL
InCHIANTI	Illumina Infinium HumanHap 550K	BeadStudio	MACH	2 543 887	MACH2QTL
LURIC	Affymetrix 6.0	Birdseed v2	MACH	2 543 887	QUICKTEST
MESA	Affymetrix 6.0	BeadStudio	HapMap1+2 IMPUTE2	2 661 456	Stata version 12
MrOS Gothenburg	Illumina Human1M-Duo/Illumina HumanOmni1-Quad	BeadStudio	MACH, MINIMAC	2 427 239	PLINK
MrOS Malmö	Illumina Omni express	BeadStudio	MACH, MINIMAC	2 515 253	PLINK
MrOS US	Illumina HumanOmni1_Quad_v1-0 B	BeadStudio	MINIMAC	2 391 250	R
RS-1	Illumina Infinium HumanHap 550K	BeadStudio	MACH	2 607 936	MACH2QTL
**Replication Cohort**					
EMAS	KASPar/Taqman	NA	NA	NA	NA

NA; not applicable

Supplemental Table 3

Characteristics of 12,774 men from 10 cohorts included in the genome-wide association study meta-analysis

	FHS	GOOD	InCHIANTI	LURIC	MESA	MrOS Gothenburg	MrOS Malmö	MrOS US	RS-1 estradiol	RS-1 estrone	EMAS
	Discovery Cohorts										Replication cohort
N	3063	929	464	2031	1194	864	895	1202	455	627	1641
Age (yrs)	49.4 (13.8)	18.9 (0.6)	67.1 (15.4)	61.9 (10.7)	62.7 (10.2)	75.2 (3.2)	75.6 (3.2)	74.1 (6.0)	68.7 (8.1)	68.9 (8.1)	59.6 (10.9)
BMI (kg/m^2)	28.3 (4.6)	22.4 (3.2)	27.1 (6.5)	27.6 (3.7)	28.0 (4.1)	26.2 (3.5)	26.5 (3.6)	27.4 (3.7)	25.7 (3.0)	25.7 (3.0)	27.7 (4.1)
E2 (pg/mL)	27.7 (9.6)	18.6 (6.1)	13.5 (5.1)	41.3 (16.9)	30.8 (11.8)	21.3 (7.6)	19.9 (7.3)	22.5 (8.5)	12.7 (6.6)	N/A	20.0 (7.1)
E1 (pg/mL)	43.1 (17.0)	22.7 (7.7)	N/A	N/A	N/A	36.7 (12.9)	30.7 (14.0)	33.0 (13.7)	N/A	24.6 (14.7)	N/A
Testosterone (ng/dl)	622 (232)	470 (149)	444 (136)	498 (203)	432 (155)	456 (169)	446 (180)	404 (163)	326 (108)	329 (113)	477 (174)
SHBG (nmol/l)	48.7 (25.3)	20.4 (7.2)	98.3 (51.8)	48.8 (26.3)	44.7 (18)	51.5 (28.2)	48.4 (21.0)	49.6 (19.8)	36.2 (13.6)	35.7 (13.4)	43 (19.4)
Serum sample	Fasting	fasting	fasting	fasting	fasting	non-fasting	non-fasting	fasting	fasting	fasting	fasting
Assay E1/E2	LC-MS	GC-MS	RIA	CLIA	RIA	GC-MS	GC-MS	GC-MS	RIA	RIA	GC-MS

Continuous parameters are given as mean (SD)

BMI, body mass index; SHBG, sex hormone-binding globulin; GC-MS, gas chromatography-mass spectroscopy; CLIA. chemiluminescence enzyme immunoassay; RIA, radioimmunoassay

N/A; not analyzed

Supplemental Table 4.

Look-up of genome-wide significant *CYP19A1* SNPs and metabolic phenotypes in men and women combined

			HOMA-IR			Fasting insulin			Fasting glucose		
**SNP**	**EA**	**FREQ**	**Effect**	**SE**	**p**	**Effect**	**SE**	p	**Effect**	**SE**	**p**
rs727479	A	0.68	-0.012	0.004	**0.004**	-0.010	0.003	**0.003**	-0.001	0.003	0.876
rs2899472	A	0.25	-0.014	0.005	**0.003**	-0.009	0.004	0.017*	-0.002	0.004	0.589
rs16964258	G	0.04	0.018	0.010	0.052	0.006	0.007	0.407	-0.017	0.007	0.024

EA = Effect Allele, *i.e.* the allele associated with increased serum E2; FREQ = Frequency of effect allele; Effect size is given per effect allele as (fasting insulin x fasting glucose)/22.5 for HOMA-IR, pmol/L for fasting insulin and mmol/L for glucose; Numbers in bold represent statistical significance after Bonferroni correction for three phenotypes; * p<0.05 after Bonferroni correction for three phenotypes. HOMA-IR n=37,000; Fasting insulin n=51,750; Fasting glucose n= 58,074 (32, 33)

Supplemental Materials and Methods - Study specific cohort information

Framingham Heart Study (FHS)

The FHS was initiated in 1948 to examine the determinants of cardiovascular disease and its risk factors (http://www.framinghamheartstudy.org/). The Original Cohort comprised 5,209 men and women, aged 28-62 years at enrollment who have undergone routine biennial examinations (1). In 1971, 5,124 Offspring of the Original Cohort participants and Offspring spouses, aged 5 to 70 years, were enrolled into the Framingham Offspring Study and have been examined approximately every 4 to 8 years (2, 3). In the 1990s, DNA was obtained for genetic studies from surviving Original cohort and Offspring participants. From 2002 to 2005, 4,095 men and women aged 20 and older with at least one parent in the Offspring cohort were enrolled in the Third Generation Cohort and DNA was obtained for genetic studies at the time of the first examination (4). Routine examinations for all FHS cohorts included a standardized physician administered medical history interview and physical examination, direct measurement of cardiovascular risk factors, laboratory assessment, and various questionnaires and noninvasive cardiovascular tests specific to the given examination cycle. Fasting serum samples from Offspring examination 7 (1998 to 2001) and Generation 3 examination 1 (2002-2005) were used to measure serum SHBG, T, E2 and E1. FHS examinations were approved by the Institutional Review Board of the Boston University Medical Center and all participants provided written informed consent.

Estrone and Estradiol assays

Blood samples were drawn in the supine position, typically between 07:30 AM and 09:30 AM after an overnight fast. Sera were aliquoted and immediately stored at -80º C, remaining frozen until the time of assay. Serum estradiol and estrone levels were measured simultaneously using LC-MS/MS after derivatization with dansyl chloride. The limit of quantitation for both hormones was 2 pg/mL. Interassay CVs for estrone were 4.5%, 7.7%, and 6.9% at estrone concentrations of 8, 77, and 209 pg/mL, respectively, and for estradiol 6.9%, 7.0%, and 4.8% at estradiol concentrations of 8, 77, and 206 pg/ml, respectively (5, 6).

Testosterone assay

Serum testosterone levels were measured by liquid chromatography tandem mass-spectrometry (LC-MS/MS) as previously described. The functional sensitivity of the testosterone assay was 2 ng/dl and the interassay coefficient of variation was 15.8%, at 12.0 ng/dL, 10.6%, at 23.5 ng/dL, 7.9%, at 48.6 ng/dL, 7.7% at 241 ng/dL, 4.4% at 532 ng/dL, and 3.3% at 1016 ng/dL respectively. As part of the Centers for Disease Control's (CDC) Testosterone Assay Harmonization Initiative, quality control samples provided by the CDC were run every three months; the bias in quality control samples with testosterone concentrations in 100 to 1000 ng/dL range was consistently less than 6% (6, 7)

SHBG assay

SHBG was measured using a two-site directed immunofluorometric assay that had a sensitivity of 0.5 nM (Delphia-Wallac, Inc., Turku, Finland) (7).

Height and weight
Height was measured without shoes using a stadiometer and was rounded to the nearest one quarter inch. Weight was measured with a calibrated balance beam scale without clothes.

Exclusions
Chemical or surgical castration and/or medications affecting sex hormones such as steroid 5-alpha reductase inhibitors, and sex hormone antagonists.

Disclosures
No disclosures

Gothenburg Osteoporosis and Obesity Determinants (GOOD) study
The Gothenburg Osteoporosis and Obesity Determinants (GOOD) study was initiated to determine both environmental and genetic factors involved in the regulation of bone and fat mass. Male study subjects from the greater Gothenburg area in Sweden were randomly selected from national population registers, contacted by telephone, and invited to participate. To be enrolled in the GOOD study, subjects had to be between 18 and 20 years of age. There were no other exclusion criteria, and 49% of the study candidates agreed to participate (n = 1068). The study was approved by the ethics committee at the University of Gothenburg. Written and oral informed consent was obtained from all study participants (8).

Estrone and Estradiol assays
A validated gas chromatography/mass spectroscopy system was used for the analysis of total E1 and E2 on frozen serum aliquots (E1: limit of detection, 8.00 pg/ml; intra-assay CV, 1.8%; interassay CV, 1.7%, E2: limit of detection, 2.00 pg/ml; intra-assay CV, 1.5%; interassay CV, 2.7%).

Testosterone assay
A validated gas chromatography/mass spectroscopy system was used for the analysis of total testosterone on frozen serum aliquots (limit of detection, 5 ng/dl; intra-assay CV, 2.9%; interassay CV, 3.4%) (9).

SHBG assay
Serum SHBG was measured using immunoradiometric assay (Orion Diagnostics, Espoo, Finland) with a limit of detection of 1.3 nmol/liter, intraassay CV of 3%, and interassay CV of 7%.

Height and weight
Height and weight were measured using standardized equipment. The CV values were <1% for these measurements.

Exclusions
Chemical or surgical castration and/or medications affecting sex hormones such as steroid 5-alpha reductase inhibitors, and sex hormone antagonists.

Disclosures
No disclosures.

InCHIANTI study
The InCHIANTI study is a population-based epidemiological study aimed at evaluating factors that influence mobility in the older population living in the Chianti region of Tuscany, Italy. Details of the study have been previously reported (10, 11). Briefly, 1616 residents were selected from the population registry of Greve in Chianti (a rural area; 11,709 residents with 19.3% of the population greater than 65 years of age) and Bagno a Ripoli (Antella village near Florence; 4,704 inhabitants, with 20.3% greater than 65 years of age). The participation rate was 90% (n= 1,453) and participants ranged between 21–102 years of age. The study protocol was approved by the Italian National Institute of Research and Care of Aging Institutional Review.

Estradiol assay
Total E2 was measured in the Laboratory of the University of Parma using ultrasensitive RIA (DSL-4800, Chematil, Angri, Italy) with a minimum detectable concentration (MDC) of 2.2 pg/ml and intra- and interassay coefficients of variation (CVs) of 8 and 10%, respectively.

Testosterone assay
Total testosterone was assayed using commercial radioimmunological kits (Diagnostic Systems Laboratories, Webster, TX). The minimal detectable concentration (MDC) was 0.08 nmol/liter; intraassay and interassay CVs for three different concentrations were 9.6, 8.1, and 7.8%, and 8.6, 9.1, and 8.4%, respectively.

SHBG assay
SHBG was measured using immunoradiometric assay (Diagnostic Products, Los Angeles, CA) with an MDC of 3.00 nmol/liter, and inter- and intraassay CV concentrations for three different concentrations were 3.7, 1.1, and 3.4% and 11.5, 10.3, and 8.7%, respectively.

Height and weight
Height and weight were measured using standardized equipment. The CV values were <1% for these measurements.

Exclusions
Exclusion criteria included chemical or surgical castration and/or medications affecting sex hormones such as steroid 5-alpha reductase inhibitors, and sex hormone antagonists. Nine individuals were excluded due to extreme E2 values (> 4 SDs).

Disclosures
No disclosures

The Ludwigshafen Risk and Cardiovascular Health (LURIC)
The Ludwigshafen Risk and Cardiovascular Health (LURIC) study is a prospective study of

more than 3,300 individuals of German ancestry in whom cardiovascular and metabolic phenotypes (CAD, MI, dyslipidemia, hypertension, metabolic syndrome and diabetes mellitus) have been defined or ruled out using standardized methodologies in all study participants. Inclusion criteria for LURIC were: German ancestry (limitation of genetic heterogeneity), clinical stability (except for acute coronary syndromes) and availability of a coronary angiogram. Exclusion criteria were: any acute illness other than acute coronary syndromes, any chronic disease where non-cardiac disease predominated and a history of malignancy within the last five years. Genome-wide analyses using the Affymetrix 6.0 have been completed in all participants. A 10-year clinical follow-up for total and cause specific mortality has been completed. The study was approved by the ethics committee at the "Landesärztekammer Rheinland-Pfalz" and was conducted in accordance with the "Declaration of Helsinki". Informed written consent was obtained from all participants.

Estradiol assay

E2 was measured using a solid-phase chemiluminescence enzyme immunoassay (Oestradiol-Immulite®, DPC Biermann GmbH, Bad Nauheim, Germany, on an Immulite autosampler until 20 Jan 1998) and a microparticle enzyme immunoassay (Oestradiol/Abbott AXYM®, Abbott GmbH, Wiesbaden, Germany, since 21 Jan 1998).

Testosterone assay

Testosterone was measured in serum using a solid-phase chemoluminescence enzyme immunoassay (Testosterone Immulite; DPC Biermann GmbH, Bad Nauheim, Germany) with an intra- and interassay coefficient of variation (CV) of 7.2 and 9.1%, respectively.

SHBG assay

SHBG was measured by a luminescence immunoassay (Roche, Basel, Switzerland) with an intra- and interassay CV of 1.3 and 2.1%, respectively.

Height and weight

Height and weight were measured using standardized equipment.

Exclusions

Exclusion criteria included chemical or surgical castration and/or medications affecting sex hormones such as steroid 5-alpha reductase inhibitors, and sex hormone antagonists.

Disclosures

Winfried März is employed with Synlab Holding Deutschland GmbH.

Multi-Ethnic Study of Atherosclerosis (MESA)

The Multi-Ethnic Study of Atherosclerosis is a 6-field center population-based study that enrolled 6,814 men and women age 45 to 85 years, without clinical cardiovascular disease from six United States communities (Baltimore, MD; Chicago, IL; Forsyth County, NC; Los Angeles County, CA; northern Manhattan, NY; and St. Paul, MN). The principal aim of MESA is to investigate subclinical cardiovascular disease and its progression. Sampling and recruitment procedures have been previously described in detail (reference below).

Adults with symptoms or history of medical or surgical treatment for cardiovascular disease were excluded. During the recruitment process, potential participants were asked about their race/ethnicity. Self-reported ethnicity was used to classify participants into groups.
This analysis included only men of European descent (12).

Estradiol assay
Estradiol was measured using an ultra-sensitive radioimmunoassay kit from Diagnostic System Laboratories (Webster, TX) at the University of Massachusetts Medical Center, Worcester, MA (limit of quantification 2.2 pg/mL, CV 10.5%) (13).

Testosterone assay
Testosterone was measured using a radioimmune assay at the University of Massachusetts Medical Center, Worcester, MA (Limit of Quantification: not provided by the laboratory, CV 12.3%).

SHBG assay
Sex hormone binding globulin (SHBG) was measured by chemiluminescent enzyme immunometric assay using Immulite kits (Diagnostic Products Corporation, Los Angeles, CA) at the University of Massachusetts Medical Center, Worcester, MA (Limit of Quantification: not provided by the laboratory, CV 9%).

Height and weight
Height and weight were measured using standardized equipment.

Exclusions
Exclusion criteria included chemical or surgical castration and/or medications affecting sex hormones such as steroid 5-alpha reductase inhibitors, and sex hormone antagonists; symptoms or history of medical or surgical treatment for cardiovascular disease; and non-availability of fasting blood sample.

Disclosures
Dhananjay Vaidya is a consultant for Consumable Science Inc.

MrOS Sweden - Gothenburg and Malmö
The Osteoporotic Fractures in Men (MrOS) study is a prospective multicenter study including older men in Sweden (3014), Hong Kong (~2000), and the United States (~6000). This study comprises a subsample of men included in the Gothenburg (n=1010) and Malmö (n= 1005) parts of the Swedish cohort (14, 15). At baseline, letters were sent to a randomly selected group of subjects (men aged 69 to 81 years old) identified in national population registers and contacted by telephone. To be eligible for the study, the participants had to be able to walk without aid, sign an informed consent, and complete a questionnaire. The inclusion rate at baseline for the Swedish part of the MrOS study was 45% (16). Exclusion criteria included chemical or surgical castration and/or medications affecting sex hormones such as steroid 5-alpha reductase inhibitors, and sex hormone antagonists. The study was approved by the ethics

committee at the University of Gothenburg and Lund. Written and oral informed consent was obtained from all study participants.

Estrone and Estradiol assays
A validated gas chromatography/mass spectroscopy system was used for the analysis of total E1 and E2 on frozen serum aliquots (E1: limit of detection, 8.00 pg/ml; intra-assay CV, 1.8%; interassay CV, 1.7%, E2: limit of detection, 2.00 pg/ml; intra-assay CV, 1.5%; interassay CV, 2.7%).

Testosterone assay
A validated gas chromatography/mass spectroscopy system was used for the analysis of total Tesstosterone on frozen serum aliquots (limit of detection, 5 ng/dl; intra-assay CV, 2.9%; interassay CV, 3.4%) (9).

SHBG assay
Serum SHBG was measured using immunoradiometric assay (Orion Diagnostics, Espoo, Finland) with a limit of detection of 1.3 nmol/liter, intraassay CV of 3%, and interassay CV of 7%.

Height and weight
Height was measured using a Harpenden stadiometer, and weight was measured by a standard balance beam or an electric scale. Two consecutive measurements of height were performed in the same session, and the average of these measurements was calculated. If there was a difference of ≥5 mm between the first two measurements, a third measurement was performed, and the average of the two values with the least mutual discrepancy was calculated.

Exclusions
Exclusion criteria included chemical or surgical castration and/or medications affecting sex hormones such as steroid 5-alpha reductase inhibitors, and sex hormone antagonists.

Disclosures
No disclosures.

The Osteoporotic Fractures in Men (MrOS) study/US
The MrOS study enrolled 5994 participants between March 2000 and April 2002. Details of the MrOS study design and recruitment have been published elsewhere (17, 18). In brief, recruitment occurred at six US clinical centers (Birmingham, AL; Minneapolis, MN; Palo Alto, CA; Pittsburgh, PA; Portland, OR; and San Diego, CA) and was accomplished primarily through mass mailings targeted to age-eligible men. Eligible participants were community-dwelling men who were at least 65 years of age, able to walk without assistance from another person, and had not had bilateral hip replacements. Written informed consent was obtained from all participants, and the Institutional Review Board at each study site approved the study.

Estrone, Estradiol and Testosterone assays

The assay method used to determine total serum testosterone, estradiol and estrone was a gas chromatograph/mass spectrometry (GCMS) assay (Taylor Technology, Princeton NJ). A combined gas chromatographic negative ionization tandem mass spectrometry (GC/NCI/MS/MS) and liquid chromatographic electrospray tandem mass spectrometry (LC/ESI/MS/MS) bioanalytical method was used to measure steroids in serum. Briefly, the analytes and their deuterated internal standards were extracted from 1.00 mL of human serum using BondElut Certify solid phase cartridges. Estradiol, estrone, and testosterone were eluted from the solid phase cartridges with ethyl acetate. The analytes underwent three separate derivatization steps: (1) reaction with pentafluorobenzoyl chloride, (2) reaction with O (2,3,4,5,6 pentafluorobenzyl) hydroxylamine hydrochloride, and (3) reaction with N-Methyl-N-(trimethylsilyl)trifluoroacetamide. Then the derivatized analytes were separated by gas chromatography using a DB 17 fused silica capillary column and detected by tandem mass spectrometry using negative ion chemical ionization. A $1/(\text{concentration})^2$ weighted least squares regression procedure was used to fit a linear function to the calibration data. The limits of detection are: for estradiol, for estrone, and for testosterone.

	Limit of detection	Intra-assay CV	Inter-assay CV
Estradiol	0.625 pg/mL	6.4%	10.1%
Estrone	1.56 pg/ml	5.2%	12.9%
Testosterone	2.50 ng/dL	2.5%	6.0%

SHBG assay

The assay method used to determine SHBG concentration used an Immulite Analyzer with chemiluminescent substrate, (Diagnostic Products Corp., Los Angeles CA). Limit of detection was 0.2 nM, intraassay CV 4.6% and interassay CV 5.8%.

Height and weight

Height and weight were measured using standardized equipment.

Exclusions

Subjects were excluded if they reported having had surgical castration or reported antiandrogen or androgen therapy.

Disclosures

No disclosures

Rotterdam Study (RS)

From 1991 to 1995 all inhabitants of Ommoord, a district of Rotterdam, The Netherlands, who were 55 years or older, were invited to participate in the Rotterdam study (RS). Genotyping information was available for 5,974 participants. All of the participants were followed for incident diseases through linkage to the general practitioner data base and record review by trained medical investigators. General practitioners', hospital records as well as death certificates were used for identification of deaths and health events through 01.01.2009 (19).

Hormone measurements
Serum levels of testosterone, E1, E2 and sex hormone-binding globulin (SHBG) were estimated in 12 batches by coated-tube or double-antibody radioimmunoassays, purchased from Diagnostic Systems Laboratories (Webster, TX, USA). For E2 estimations, the ultrasensitive system was used. The results of these assays were compared with the results obtained with other commercial immunoassays, which in turn had been validated by comparison with in-house immunoassays, making use of steroid extraction and purification by column chromatography (testosterone (20), E2 (21) and E1 (22)). The same procedure was used for SHBG, where the in-house method used ammonium sulphate precipitation (23). Correlation coefficients varied from 0.925 to 0.980. The sensitivities of the assays, defined as the value representing the blank plus twice the standard deviation of the blank, were 4.8 pmol/l for E2 (24), 0.28 nmol/l for testosterone and 5 nmol/l for SHBG. Because of the relatively small volumes of serum available, all values reported are single-sample estimations. Intra-assay coefficients of variation, determined on the basis of duplicate results of internal quality control (QC) serum pools with three different levels of each analyte, were below 15% for all assays, with the exception of E2 (18%) and E1 (21%). Since inter-assay variations were relatively large (20–30%, with the exception of testosterone (19%) and SHBG (14%)), the results of all batches were normalized by multiplying all concentrations within a batch by a factor, a method which equalized results for the internal quality-control pools. This was considered justified because the results of these pools and the mean results for male and female sera in each assay batch showed very similar patterns (25).

Height and weight
Height and weight were measured using standardized equipment.

Exclusions
Exclusion criteria included chemical or surgical castration and/or medications affecting sex hormones such as steroid 5-alpha reductase inhibitors, and sex hormone antagonists.

European Male Ageing Study (EMAS)
The EMAS is a prospective, population-based study of ageing in middle aged and elderly European men. Men were recruited from population based sampling frames in 8 centres: Florence (Italy), Leuven (Belgium), Lodz (Poland), Malmo (Sweden) Manchester (UK) Santiago del Compostella (Spain), Szeged (Hungary), Tartu (Estonia). Details regarding recruitment, response rates and assessments have been described previously (26). Stratified random sampling was performed in each centre with the aim of recruiting one hundred men in each of four 10-year age bands: 40-49 years, 50-59 years, 60-69 years, and 70-79 years. Subjects were invited by letter to attend for a clinical assessment. A single fasting morning (before 10 a.m.) venous blood sample was obtained from all subjects. Serum was separated immediately after phlebotomy and stored at -80°C until assay at the end of the baseline study. Ethical approval for the study was obtained in accordance with local institutional requirements in each centre (26).

Estrone and Estradiol assays
Measurement of E2 was carried out by gas chromatography mass spectrometry (GC-MS) as described in Labrie et al (27, 28). The lower limit of E2 quantitation was 7.34 pmol/L. The coefficients of variation of E2 measurements were 3.5% within runs and 3.7% between runs.

Testosterone assay
Measurement of T was carried out by GC-MS as described in Labrie et al. (27, 28). The lower limit of T quantitation was 0.17 nmol/L. The coefficients of variation of T measurements were 2.9% within runs and 3.4% between runs.

SHBG assay
SHBG was measured by the Modular E170 platform electrochemiluminescence immunoassay (Roche Diagnostics, Mannheim, Germany). Within- and between-assay coefficients of variation were 1.70 and 3.18%, respectively. The detection limit was 0.35 nmol/L.

Height and weight
Height and weight were measured in a standardized manner in the standing position.

Exclusions
From a total of 3,369 participants, men with missing genotype or phenotype data were excluded, giving 1,641 men in the analysis sample.

Disclosures
Ilpo T Huhtaniemi received grants from Ferring Pharmaceutical, and did consultation for Novartis and Takeda

Acknowledgements

FHS
The authors thank the Framingham Heart Study participants and staff. The Framingham Heart Study phenotype-genotype analyses were supported by the National Institute of Aging (Genetics of Reproductive Life Period and Health Outcomes, R21AG032598; JMM, KL and R01AG29451 JMM, KL) and the National Institute of Arthritis Musculoskeletal and Skin Diseases (R01 AR41398 DPK). The Framingham Heart Study of the National Heart Lung and Blood Institute of the National Institutes of Health and Boston University School of Medicine was supported by the National Heart, Lung and Blood Institute's Framingham Heart Study Contract No. N01-HC-25195 and its contract with Affymetrix, Inc for genotyping services (Contract No. N02-HL-6-4278). Analyses reflect intellectual input and resource development from the Framingham Heart Study investigators participating in the SNP Health Association Resource (SHARe) project. A portion of this research was conducted using the Linux Cluster for Genetic Analysis (LinGA-II) funded by the Robert Dawson Evans Endowment of the Department of Medicine at Boston University School of Medicine and Boston Medical Center. The hormone measurements in the FHS were supported by NIH grants 5R01DK092938-04 and 1R01AG31206-01 to Shalender Bhasin.

GOOD
Financial support was received from the Swedish Research Council, the Swedish Foundation for Strategic Research, the ALF/LUA research grant in Gothenburg, the Lundberg Foundation, the Torsten and Ragnar Söderberg's Foundation, the Novo Nordisk Foundation, and the European Commission grant HEALTH-F2-2008-201865-GEFOS.

InCHIANTI
The InCHIANTI study baseline (1998-2000) was supported as a "targeted project" (ICS110.1/RF97.71) by the Italian Ministry of Health and in part by the U.S. National Institute on Aging (Contracts: 263 MD 9164 and 263 MD 821336); the InCHIANTI Follow-up 1 (2001-2003) was funded by the U.S. National Institute on Aging (Contracts: N.1-AG-1-1 and N.1-AG-1-2111); the InCHIANTI Follow-ups 2 and 3 studies (2004-2010) were financed by the U.S. National Institute on Aging (Contract: N01-AG-5-0002);supported in part by the Intramural research program of the National Institute on Aging, National Institutes of Health, Baltimore, Maryland. JRBP is supported by the Wellcome Trust as a Sir Henry Wellcome Fellow (092447/B/10/Z).

LURIC
We extend our appreciation to the participants of the LURIC study and thank the LURIC study team who were either temporarily or permanently involved in patient recruitment as well as sample and data handling, in addition to the laboratory staff at the Ludwigshafen General Hospital and the Universities of Freiburg and Ulm, Germany. LURIC was supported by the 7th Framework Program (AtheroRemo, grant agreement number 201668 and RiskyCAD, grant agreement number 305739) of the EU and by the INTERREG-IV-Oberrhein-Program (Project A28, Genetic mechanisms of cardiovascular diseases) with support from the European Regional Development Fund (ERDF) and the Wissenschaftsoffensive TMO.

MESA
MESA and the MESA SHARe project are conducted and supported by the National Heart, Lung, and Blood Institute (NHLBI) in collaboration with MESA investigators. Support for MESA is provided by contracts HHSN268201500003I, N01-HC-95159, N01-HC-95160, N01-HC-95161, N01-HC-95162, N01-HC-95163, N01-HC-95164, N01-HC-95165, N01-HC-95166, N01-HC-95167, N01-HC-95168, N01-HC-95169, UL1-TR-000040, UL1-TR-001079, UL1-TR-001420, UL1-TR-001881, and DK063491. Funding support for the sex hormone dataset was provided by grants HL074406 and HL074338.

MrOS Sweden
The MrOS Sweden study is supported by the Swedish Research Council, the Swedish Foundation for Strategic Research, the ALF/LUA research grant in Gothenburg, the Lundberg Foundation, Knut and Alice Wallenberg Foundation, the Torsten and Ragnar Söderberg's Foundation and the Novo Nordisk Foundation.

MrOS US
The Osteoporotic Fractures in Men (MrOS) Study is supported by National Institutes of Health funding. The following institutes provide support: the National Institute on Aging

(NIA), the National Institute of Arthritis and Musculoskeletal and Skin Diseases (NIAMS), the National Center for Advancing Translational Sciences (NCATS), and NIH Roadmap for Medical Research under the following grant numbers: U01 AG027810, U01 AG042124, U01 AG042139, U01 AG042140, U01 AG042143, U01 AG042145, U01 AG042168, U01 AR066160, UL1 TR000128, and RC2 AR058973.

RS

The generation and management of GWAS genotype data for the Rotterdam Study (RS I, RS II, RS III) was executed by the Human Genotyping Facility of the Genetic Laboratory of the Department of Internal Medicine, Erasmus MC, Rotterdam, The Netherlands. The GWAS datasets are supported by the Netherlands Organisation of Scientific Research NWO Investments (nr. 175.010.2005.011, 911-03-012), the Genetic Laboratory of the Department of Internal Medicine, Erasmus MC, the Research Institute for Diseases in the Elderly (014-93-015; RIDE2), the Netherlands Genomics Initiative (NGI)/Netherlands Organisation for Scientific Research (NWO) Netherlands Consortium for Healthy Aging (NCHA), project nr. 050-060-810. We thank Pascal Arp, Mila Jhamai, Marijn Verkerk, Lizbeth Herrera and Marjolein Peters, MSc, and Carolina Medina-Gomez, MSc, for their help in creating the GWAS database, and Karol Estrada, PhD, Yurii Aulchenko, PhD, and Carolina Medina-Gomez, MSc, for the creation and analysis of imputed data. The Rotterdam Study is funded by Erasmus Medical Center and Erasmus University, Rotterdam, Netherlands Organization for the Health Research and Development (ZonMw), the Research Institute for Diseases in the Elderly (RIDE), the Ministry of Education, Culture and Science, the Ministry for Health, Welfare and Sports, the European Commission (DG XII), and the Municipality of Rotterdam. The authors are grateful to the study participants, the staff from the Rotterdam Study and the participating general practitioners and pharmacists.

EMAS

The EMAS is funded by the Commission of the European Communities Fifth Framework Programme "Quality of Life and Management of Living Resources" Grant QLK6-CT-2001-00258 and supported by funding from Arthritis Research UK. The EMAS Principal Investigator is Professor Frederick Wu, MD; Centre for Endocrinology and Diabetes, The University of Manchester, Manchester UK. The "EMAS Study Group" consists of the following people: Gyorgy Bartfai, Steven Boonen, Felipe Casanueva, Joseph D Finn, Gianni Forti, Aleksander Giwercman, Thang S Han, Kate L Holliday, Ilpo T Huhtaniemi, Krzysztof Kula, Michael EJ Lean, Terence W O'Neill, Neil Pendleton, Margus Punab, Stephen R Pye, Wendy Thomson, Dirk Vanderschueren, and Frederick CW Wu. The authors wish to thank the men who participated in the eight countries and the research/nursing staff in the eight centres: C Pott, Manchester, E Wouters, Leuven, M Nilsson, Malmö, M del Mar Fernandez, Santiago de Compostela, M Jedrzejowska, Lodz, H-M Tabo, Tartu, A Heredi, Szeged for their data collection and C Moseley, Manchester for data entry and project coordination. Dr Vanderschueren is a senior clinical investigator supported by the Clinical Research Fund of the University Hospitals Leuven, Belgium.

References

1. Dawber TR, Meadors GF, Moore FE, Jr. Epidemiological approaches to heart disease: the Framingham Study. *Am J Public Health Nations Health*. 1951;41(3):279-81.
2. Feinleib M, Kannel WB, Garrison RJ, McNamara PM, Castelli WP. The Framingham Offspring Study. Design and preliminary data. *Prev Med*. 1975;4(4):518-25.
3. Kannel WB, Feinleib M, McNamara PM, Garrison RJ, Castelli WP. An investigation of coronary heart disease in families. The Framingham offspring study. *Am J Epidemiol*. 1979;110(3):281-90.
4. Splansky GL, Corey D, Yang Q, Atwood LD, Cupples LA, Benjamin EJ, D'Agostino RB, Sr., Fox CS, Larson MG, Murabito JM, O'Donnell CJ, Vasan RS, Wolf PA, Levy D. The Third Generation Cohort of the National Heart, Lung, and Blood Institute's Framingham Heart Study: design, recruitment, and initial examination. *Am J Epidemiol*. 2007;165(11):1328-35.
5. Jasuja GK, Travison TG, Davda M, Murabito JM, Basaria S, Zhang A, Kushnir MM, Rockwood AL, Meikle W, Pencina MJ, Coviello A, Rose AJ, D'Agostino R, Vasan RS, Bhasin S. Age trends in estradiol and estrone levels measured using liquid chromatography tandem mass spectrometry in community-dwelling men of the Framingham Heart Study. *J Gerontol A Biol Sci Med Sci*. 2013;68(6):733-40.
6. Snyder PJ, Bhasin S, Cunningham GR, Matsumoto AM, Stephens-Shields AJ, Cauley JA, Gill TM, Barrett-Connor E, Swerdloff RS, Wang C, Ensrud KE, Lewis CE, Farrar JT, Cella D, Rosen RC, Pahor M, Crandall JP, Molitch ME, Cifelli D, Dougar D, Fluharty L, Resnick SM, Storer TW, Anton S, Basaria S, Diem SJ, Hou X, Mohler ER, 3rd, Parsons JK, Wenger NK, Zeldow B, Landis JR, Ellenberg SS. Effects of Testosterone Treatment in Older Men. *N Engl J Med*. 2016;374(7):611-24.
7. Bhasin S, Pencina M, Jasuja GK, Travison TG, Coviello A, Orwoll E, Wang PY, Nielson C, Wu F, Tajar A, Labrie F, Vesper H, Zhang A, Ulloor J, Singh R, D'Agostino R, Vasan RS. Reference ranges for testosterone in men generated using liquid chromatography tandem mass spectrometry in a community-based sample of healthy nonobese young men in the Framingham Heart Study and applied to three geographically distinct cohorts. *J Clin Endocrinol Metab*. 2011;96(8):2430-9.
8. Lorentzon M, Swanson C, Andersson N, Mellstrom D, Ohlsson C. Free testosterone is a positive, whereas free estradiol is a negative, predictor of cortical bone size in young Swedish men: the GOOD study. *J Bone Miner Res*. 2005;20(8):1334-41.
9. Vandenput L, Labrie F, Mellstrom D, Swanson C, Knutsson T, Peeker R, Ljunggren O, Orwoll E, Eriksson AL, Damber JE, Ohlsson C. Serum levels of specific glucuronidated androgen metabolites predict BMD and prostate volume in elderly men. *J Bone Miner Res*. 2007;22(2):220-7.
10. Ferrucci L, Bandinelli S, Benvenuti E, Di Iorio A, Macchi C, Harris TB, Guralnik JM. Subsystems contributing to the decline in ability to walk: bridging the gap between epidemiology and geriatric practice in the InCHIANTI study. *J Am Geriatr Soc*. 2000;48(12):1618-25.
11. Melzer D, Perry JR, Hernandez D, Corsi AM, Stevens K, Rafferty I, Lauretani F, Murray A, Gibbs JR, Paolisso G, Rafiq S, Simon-Sanchez J, Lango H, Scholz S, Weedon MN, Arepalli S, Rice N, Washecka N, Hurst A, Britton A, Henley W, van de Leemput J, Li R, Newman AB, Tranah G, Harris T, Panicker V, Dayan C, Bennett A, McCarthy MI, Ruokonen

A, Jarvelin MR, Guralnik J, Bandinelli S, Frayling TM, Singleton A, Ferrucci L. A genome-wide association study identifies protein quantitative trait loci (pQTLs). *PLoS Genet*. 2008;4(5):e1000072.
12. Bild DE, Bluemke DA, Burke GL, Detrano R, Diez Roux AV, Folsom AR, Greenland P, Jacob DR, Jr., Kronmal R, Liu K, Nelson JC, O'Leary D, Saad MF, Shea S, Szklo M, Tracy RP. Multi-Ethnic Study of Atherosclerosis: objectives and design. *Am J Epidemiol*. 2002;156(9):871-81.
13. Ouyang P, Vaidya D, Dobs A, Golden SH, Szklo M, Heckbert SR, Kopp P, Gapstur SM. Sex hormone levels and subclinical atherosclerosis in postmenopausal women: the Multi-Ethnic Study of Atherosclerosis. *Atherosclerosis*. 2009;204(1):255-61.
14. Mellstrom D, Johnell O, Ljunggren O, Eriksson A, Lorentzon M, Mallmin H, Holmberg A, Redlund-Johnell I, Orwoll E, Ohlsson C. Free Testosterone is an Independent Predictor of BMD and Prevalent Fractures in Elderly Men - MrOs Sweden. *J Bone Miner Res*. 2006;21((4)):529-35.
15. Ohlsson C, Mellstrom D, Carlzon D, Orwoll E, Ljunggren O, Karlsson MK, Vandenput L. Older men with low serum IGF-1 have an increased risk of incident fractures: the MrOS Sweden study. *J Bone Miner Res*. 2011;26(4):865-72.
16. Mellstrom D, Vandenput L, Mallmin H, Holmberg AH, Lorentzon M, Oden A, Johansson H, Orwoll ES, Labrie F, Karlsson MK, Ljunggren O, Ohlsson C. Older men with low serum estradiol and high serum SHBG have an increased risk of fractures. *J Bone Miner Res*. 2008;23(10):1552-60.
17. Blank JB, Cawthon PM, Carrion-Petersen ML, Harper L, Johnson JP, Mitson E, Delay RR. Overview of recruitment for the osteoporotic fractures in men study (MrOS). *Contemp Clin Trials*. 2005;26(5):557-68.
18. Orwoll E, Blank JB, Barrett-Connor E, Cauley J, Cummings S, Ensrud K, Lewis C, Cawthon PM, Marcus R, Marshall LM, McGowan J, Phipps K, Sherman S, Stefanick ML, Stone K. Design and baseline characteristics of the osteoporotic fractures in men (MrOS) study--a large observational study of the determinants of fracture in older men. *Contemp Clin Trials*. 2005;26(5):569-85.
19. Hofman A, Brusselle GG, Darwish Murad S, van Duijn CM, Franco OH, Goedegebure A, Ikram MA, Klaver CC, Nijsten TE, Peeters RP, Stricker BH, Tiemeier HW, Uitterlinden AG, Vernooij MW. The Rotterdam Study: 2016 objectives and design update. *Eur J Epidemiol*. 2015;30(8):661-708.
20. Verjans HL, Cooke BA, de Jong FH, de Jong CM, van der Molen HJ. Evaluation of a radioimmunoassay for testosterone estimation. *J Steroid Biochem*. 1973;4(6):665-76.
21. de Jong FH, Hey AH, van der Molen HJ. Effect of gonadotrophins on the secretion of oestradiol- and testosterone by the rat testis. *J Endocrinol*. 1973;57(2):277-84.
22. Van Landeghem AA, Poortman J, Deshpande N, Di Martino L, Tarquini A, Thijssen JH, Schwarz F. Plasma concentration gradient of steroid hormones across human mammary tumours in vivo. *J Steroid Biochem*. 1981;14(8):741-7.
23. de Jong FH, Oishi K, Hayes RB, Bogdanowicz JF, Raatgever JW, van der Maas PJ, Yoshida O, Schroeder FH. Peripheral hormone levels in controls and patients with prostatic cancer or benign prostatic hyperplasia: results from the Dutch-Japanese case-control study. *Cancer Res*. 1991;51(13):3445-50.
24. Goderie-Plomp HW, van der Klift M, de Ronde W, Hofman A, de Jong FH, Pols HA. Endogenous sex hormones, sex hormone-binding globulin, and the risk of incident vertebral

fractures in elderly men and women: the Rotterdam Study. *J Clin Endocrinol Metab*. 2004;89(7):3261-9.
25. Hak AE, Witteman JC, de Jong FH, Geerlings MI, Hofman A, Pols HA. Low levels of endogenous androgens increase the risk of atherosclerosis in elderly men: the Rotterdam study. *J Clin Endocrinol Metab*. 2002;87(8):3632-9.
26. Lee DM, O'Neill TW, Pye SR, Silman AJ, Finn JD, Pendleton N, Tajar A, Bartfai G, Casanueva F, Forti G, Giwercman A, Huhtaniemi IT, Kula K, Punab M, Boonen S, Vanderschueren D, Wu FC. The European Male Ageing Study (EMAS): design, methods and recruitment. *Int J Androl*. 2009;32(1):11-24.
27. Labrie F, Belanger A, Belanger P, Berube R, Martel C, Cusan L, Gomez J, Candas B, Castiel I, Chaussade V, Deloche C, Leclaire J. Androgen glucuronides, instead of testosterone, as the new markers of androgenic activity in women. *J Steroid Biochem Mol Biol*. 2006;99(4-5):182-8.
28. Labrie F, Belanger A, Belanger P, Berube R, Martel C, Cusan L, Gomez J, Candas B, Chaussade V, Castiel I, Deloche C, Leclaire J. Metabolism of DHEA in postmenopausal women following percutaneous administration. *J Steroid Biochem Mol Biol*. 2007;103(2):178-88.
